# Effects of Pore Connectivity on Water Adsorption in Metal–Organic Frameworks

**DOI:** 10.1021/acs.langmuir.6c03373

**Published:** 2026-08-11

**Authors:** Yen-Ju Chu, Li-Chiang Lin

**Affiliations:** Department of Chemical Engineering, 33561 National Taiwan University, No. 1, Sec. 4, Roosevelt Road, Taipei 106319, Taiwan

## Abstract

Atmospheric water harvesting (AWH) has emerged as a promising solution to help address global freshwater scarcity. Metal–organic frameworks (MOFs) are premier adsorbent candidates for their highly tunable nature through combinations of metal nodes and organic linkers. Ideally, they should exhibit an “S-shaped” isotherm, featuring large water uptake that can be released within a narrow humidity range. While the effects of pore dimension and chemical affinity on their adsorption properties have been studied, the influence of spatial pore connectivity remains largely unexplored. To this end, this study investigates the role of topological connectivity in their water adsorption properties using flat-histogram Monte Carlo simulations. Specifically, two representative types of MOFs (i.e., one with connected cages and another with individual channels) are studied. Moreover, a strategic pore-blocking approach is employed to systematically modulate their structural connectivity without altering local host–adsorbate interactions. Simulation results show that enhanced spatial connectivity fundamentally promotes cooperative adsorption, notably shifting the step pressure toward a lower value. Grand potential free energy and configurational analyses further reveal that interconnected pores facilitate the formation of a more stable condensed phase with extended hydrogen-bonding networks. Interestingly, in MOFs with effectively isolated one-dimensional channels, minor interpore associative behaviors through long-range Coulombic interactions can still persist, exerting a non-negligible influence on their water adsorption behavior. Comparative simulations for nonpolar gases also confirm that this connectivity-driven step shift is a unique characteristic of strongly associated molecules like water. Overall, this work demonstrates that pore connectivity is a critical structural parameter dictating water phase behavior, offering key insights into the rational design of highly efficient AWH adsorbents.

## Graphical abstract

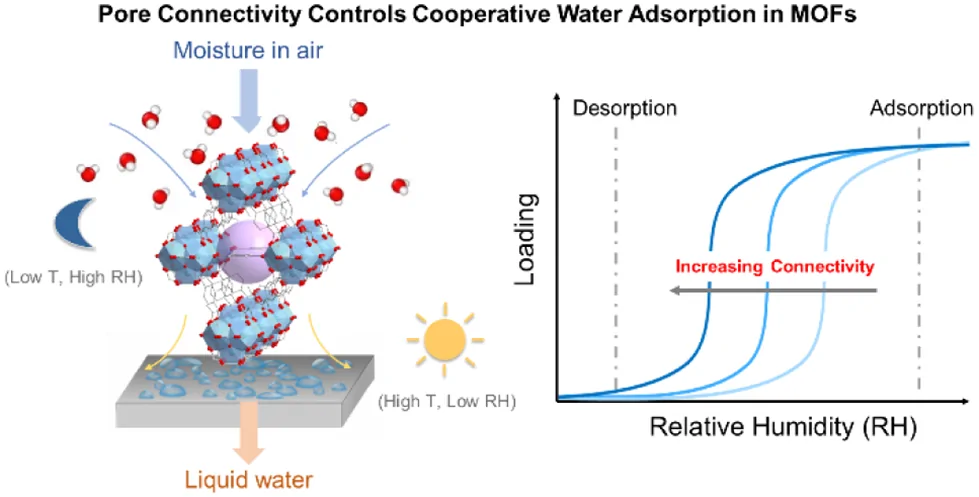

## 1 Introduction

Freshwater scarcity has become an increasingly critical global challenge, with approximately four billion people (i.e., nearly two-thirds of the world’s population) experiencing severe water shortages.^1,2^ Atmospheric water harvesting (AWH) has emerged as a promising complementary approach, particularly for inland arid regions where seawater desalination is constrained by its high energy demand and dependence on access to seawater. Among the various AWH strategies, sorption-driven water harvesting is especially attractive because it can capture water vapor from dry air and release the adsorbed water using low-grade thermal or solar energy.^3–9^

Effective sorbents for AWH should exhibit a large working capacity, preferably through an S-shaped isotherm with a sharp change in water uptake over a narrow range of relative humidity, together with a low regeneration temperature and high cycling stability. Conventional sorbents such as silica gels and zeolites are limited by relatively low water uptake capacities or high regeneration energy requirements.^10–12^ In contrast, metal–organic frameworks (MOFs),^13–15^ constructed from metal nodes and organic linkers, offer highly tunable pore environments and water adsorption properties, making them promising candidates for efficient AWH.^16,17^

To optimize the performance of MOFs for AWH, extensive research has been carried out to explore how chemical and geometric structural modifications dictate their water adsorption behaviors.^12,18,19^ In terms of chemical composition, researchers have been attempting to alter the metal nodes or introduce specific functional groups to tune the hydrophilicity and hydrophobicity of the pore surfaces. For instance, substituting the central metal ions (e.g., Mg, Ni, Co, Zn) in the MOF-74 series,^20–22^ or grafting hydrophilic (e.g., −NH_2_, −OH) and hydrophobic (e.g., −CH_3_) functional groups onto the organic linkers,^23,24^ can effectively modulate the initial water-binding strength and shift the relative humidity at which the adsorption step occurs. Efforts have also centered on the variation of secondary building units (SBUs) and the expansion of pore dimensions. By changing the coordination modes of SBUs or elongating the organic linkers, the pore size and surface area of MOFs can be controlled, which in turn determines the total water uptake capacity and the thermodynamic conditions required for capillary condensation.^17,25^ However, while “chemical affinity” and “pore size” have been investigated, recent studies have begun to investigate another key factor that may govern the water adsorption mechanism: pore connectivity. For example, Frank et al. demonstrated that even with identical zirconium-based SBUs, differences in structural connectivity can lead to entirely distinct water filling pathways.^26^ Furthermore, a large-scale thermodynamic analysis by Shih et al. showed that the dimensionality of MOFs may dictate whether the adsorption isotherm exhibits a desired S-shaped step.^27^ This perspective is also supported by Xu et al., whose study reported that the connectivity of favorable water adsorption sites in MOFs, specifically the formation of so-called continuous adsorption channels (CACs), can influence their hydrophilicity.^28^ Collectively, these findings highlight pore connectivity as an important descriptor governing cooperative water adsorption beyond conventional considerations such as pore size and surface chemistry. However, previous connectivity analyses have primarily compared different MOF structures, in which variations in connectivity are often accompanied by simultaneous changes in framework chemistry, pore size, pore shape, and framework–water interactions. Consequently, the isolated influence of pore connectivity on the thermodynamics and molecular mechanisms of water adsorption remains insufficiently understood.

Herein, this work employs flat histogram Monte Carlo simulations to evaluate water adsorption in porous materials with varying pore connectivity. Specifically, the role of pore connectivity in water adsorption is explored by studying two representative types of MOFs: one featuring well-connected cages and the other consisting of isolated channels. The adsorption behavior within each framework as well as its individual cage or channel is first examined to probe the effect of pore connectivity. A series of structural configurations with varied pore accessibilities via selective pore blocking, ranging from a fully interconnected framework to highly confined pore environments, are also studied for a more systematic investigation. Importantly, this pore-blocking strategy enables the accessible pore-to-pore connections to be systematically varied within the same MOF framework while preserving framework–water interactions. It therefore allows the influence of pore connectivity to be examined independently of variations in framework chemistry and pore geometry. Moreover, to highlight the unique nature of water for its strong intermolecular association, we also explore the influence of pore connectivity on the adsorption of several widely studied, nonpolar gases including methane, carbon dioxide, and nitrogen for comparison. Overall, the outcomes of this study provide insights into how connectivity uniquely controls the adsorption of water in MOFs, facilitating the future design of AWH adsorbents.

## 2 Materials and Methods

To compute the adsorption isotherm of water in MOFs, the NVT+W method is employed. This method is a flat-histogram Monte Carlo variant originally introduced by Smit and coworkers.^29,30^ While grand canonical Monte Carlo (GCMC) has been the widely adopted technique for simulating gaseous adsorption, it may encounter convergence issues for water. These stem from poor sampling of water cluster formation/removal.^31^ The NVT+W method circumvents these limitations by sampling each macrostate equally, as detailed below, and has been shown to more reliably capture water adsorption behavior in MOFs.^31–33^ Herein, to elucidate how structural connectivity dictates water adsorption, two representative types of MOF frameworks (i.e., mainly MOF-806 and LUDKOM) with contrasting pore architectures are investigated. Moreover, building upon these systems, model systems generated through strategic blocking of selected cages or channels are also employed to systematically modulate their connectivity without modifying the intrinsic framework–water interactions. Molecular simulations are also conducted to study the adsorption of several small nonpolar molecules in these systems for comparison.

### 2.1 Structures with Systematically Varied Pore Connectivity

Two representative MOF structures with distinctly different pore topologies are primarily studied as model systems in this study. Specifically, MOF-806 featuring interconnected cages is chosen to represent efficient pore communication, while LUDKOM possessing independent channels is used to represent isolated adsorption environments. Figure 1a visualizes the MOF-806 framework, whose structural data are adopted from Furukawa et al.^17^ This structure contains a central large pore surrounded by eight smaller pores, forming a three-dimensional interconnected pore network. According to geometric analysis using Zeo++ ^34–36^ for determining the pore size distribution (PSD) as shown in Figure S1a, the diameter of the large pore is approximately 12.8 Å, while each of the surrounding small pores has a diameter of about 10.0 Å. Importantly, there is no steric hindrance between the large and small pores, allowing water molecules to move freely throughout the framework. By contrast, Figure 1b illustrates the LUDKOM framework based on structural data reported by McKellar et al.^37^ It is a one-dimensional channel structure, having two distinct channels with pore diameters of approximately 6.8 Å and 6.1 Å. These values are likewise obtained from its two dominant PSD peaks as shown in Figure S1b. Adsorbed water molecules are confined within individual channels and cannot migrate to neighboring ones, leading to adsorption behavior that is primarily governed by local channel environments.

**Figure 1:**
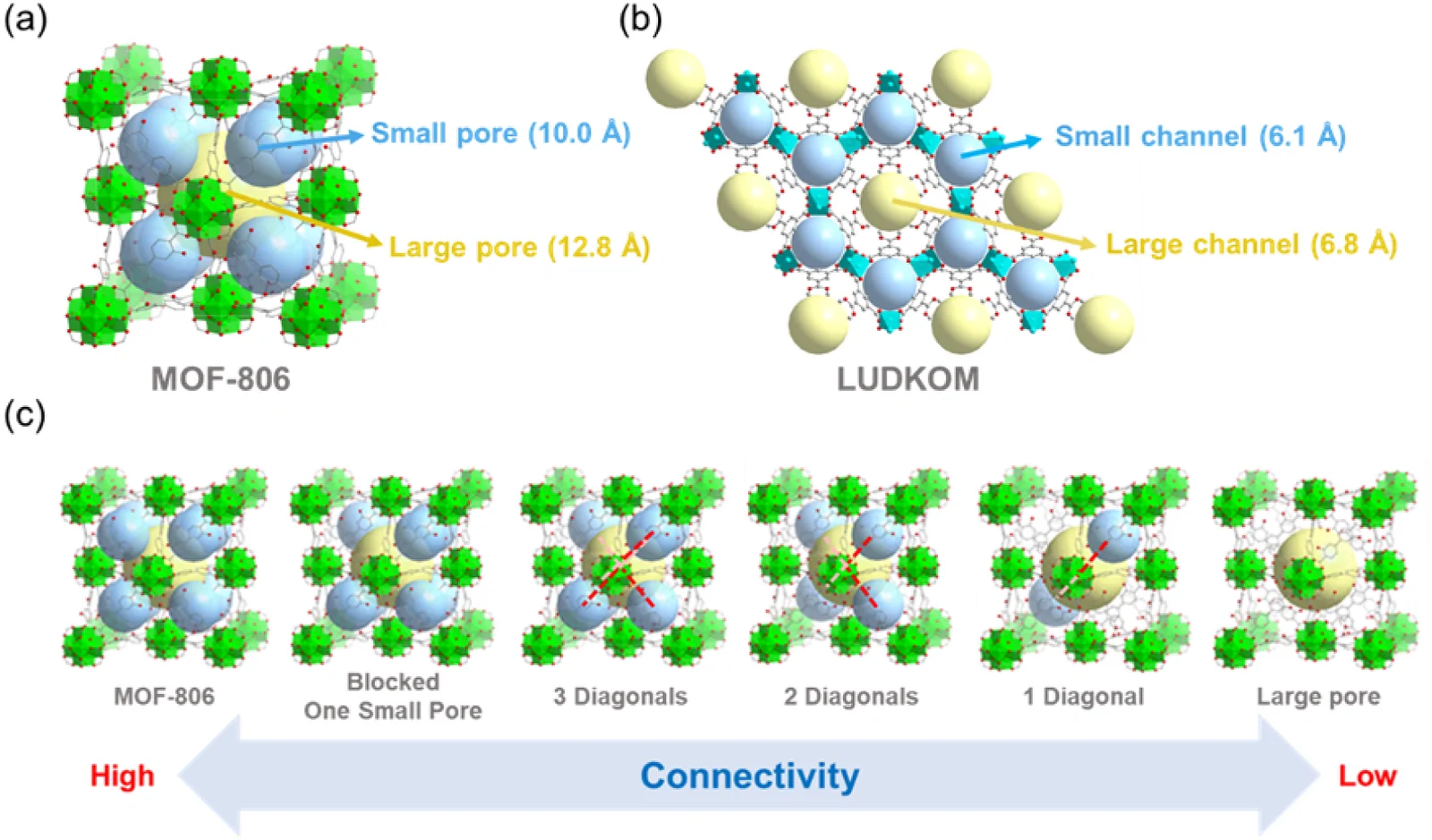
(a) Structure of MOF-806 featuring a three-dimensional interconnected pore network. The yellow sphere represents the central large pore, while the blue spheres denote the surrounding small pores. (b) Structure of LUDKOM with one-dimensional channels. The yellow and blue spheres represent the large and small channels, respectively. (c) Modified MOF-806 structures with systematically varied pore connectivity, generated by introducing blocking spheres that progressively restrict pore accessibility.

Aside from the adsorption of water in these two distinct representative frameworks, adsorption in each individual pore or channel is also investigated. This is done by a strategic blocking strategy employed in molecular simulations to restrict molecular insertions to specific pores or channels by blocking the others. It should be noted that the isolated large pore and isolated small pore systems retain all pores or channels of the corresponding type within the original simulation cell, while the other type is blocked. For MOF-806, the former includes a single large pore while the latter contains eight small pores. Moreover, a series of modified MOF-806 structures with progressively varied pore connectivity is constructed to further examine the effect of connectivity, as illustrated in Figure 1c. With the fully accessible structure as the starting point, the connectivity between the central large pore and its surrounding small pores is progressively restricted by introducing blocking spheres. Specifically, the generated models include structures in which (i) only one small pore is blocked, (ii) three diagonal connections are retained (i.e., two small pores along a given diagonal direction are blocked), (iii) two diagonal connections are retained, and (iv) only one diagonal connection is retained. They are respectively denoted as (i) blocked one small pore, (ii) 3 diagonals, (iii) 2 diagonals, and (iv) 1 diagonal. These configurations correspondingly contain seven, six, four, and two accessible small pores surrounding the central large pore, respectively, compared with eight in the fully accessible structure. The number of accessible small pores provides a quantitative description of the local connectivity, whereas the diagonal labels distinguish the spatial arrangements of the retained pore connections. Such diagonal notation has also been adopted in a prior study by Choi et al.^38^ Collectively, these systems provide a comprehensive platform to examine the impact of pore connectivity on water adsorption, enabling direct comparison between fully interconnected networks, partially restricted structures, and effectively isolated pore environments. Note that, to obtain the needed blocking spheres employed in molecular simulations, the pore space accessible to water molecules is first characterized using also the Zeo++ ^34–36^ package with a spherical probe of 2.65 Å in diameter (i.e., the kinetic diameter of water molecules),^39^ followed by generating a set of filling spheres to occupy each distinct cage or channel. Those filling spheres associated with the pore domains intended to be excluded are selected as blocking pockets. Multiple filling spheres are used collectively to occupy each excluded pore region, and any trial insertion that places a water molecule within one of these blocking spheres is rejected, thereby preventing water molecules from accessing the selected pore space.

### 2.2 NVT+W Simulations

The practical implementation of the above-mentioned NVT+W approach involves executing a set of independent canonical (NVT) simulations for each possible loading macrostate *N* that ranges from an empty framework to a maximum loading (*N_max_*). To ensure that all favorable high-density macrostates are included in the sampling, *N_max_* is set to be slightly higher than the theoretical geometric saturation loading estimated using the accessible pore volume and the density of liquid water at 298 K. Within each NVT simulation, trial Widom insertion and deletion moves are also carried out on the fly. While these so-called “ghost” insertion and deletion moves will never be accepted, their acceptance probabilities per grand canonical acceptance criteria against a reference chemical potential *μ* and temperature *T* are recorded in a transition matrix (i.e., denoted as the C-matrix). Once the C-matrix converges, it is normalized to yield unbiased transition probabilities between neighboring macrostates. Herein, convergence is assessed by monitoring the stability of the *C*(*N*,*N* – 1)/*C*(*N*,*N* + 1) ratio, and each simulation generally requires 200,000 initialization cycles followed by 100,000–200,000 production cycles. These transition probabilities are then used to construct the macrostate probability distribution (MPD), Π­(*N*; *μ*, *V*, *T*), through the principle of detailed balance. The resulting MPD can be further analytically reweighted to other chemical potential *μ*′. Finally, the equilibrium adsorption loading is determined as the MPD-weighted average number of adsorbed molecules. A schematic illustration of the NVT+W workflow with related equations is shown in Figure 2. More details are provided in the Supporting Information (SI).

**Figure 2:**
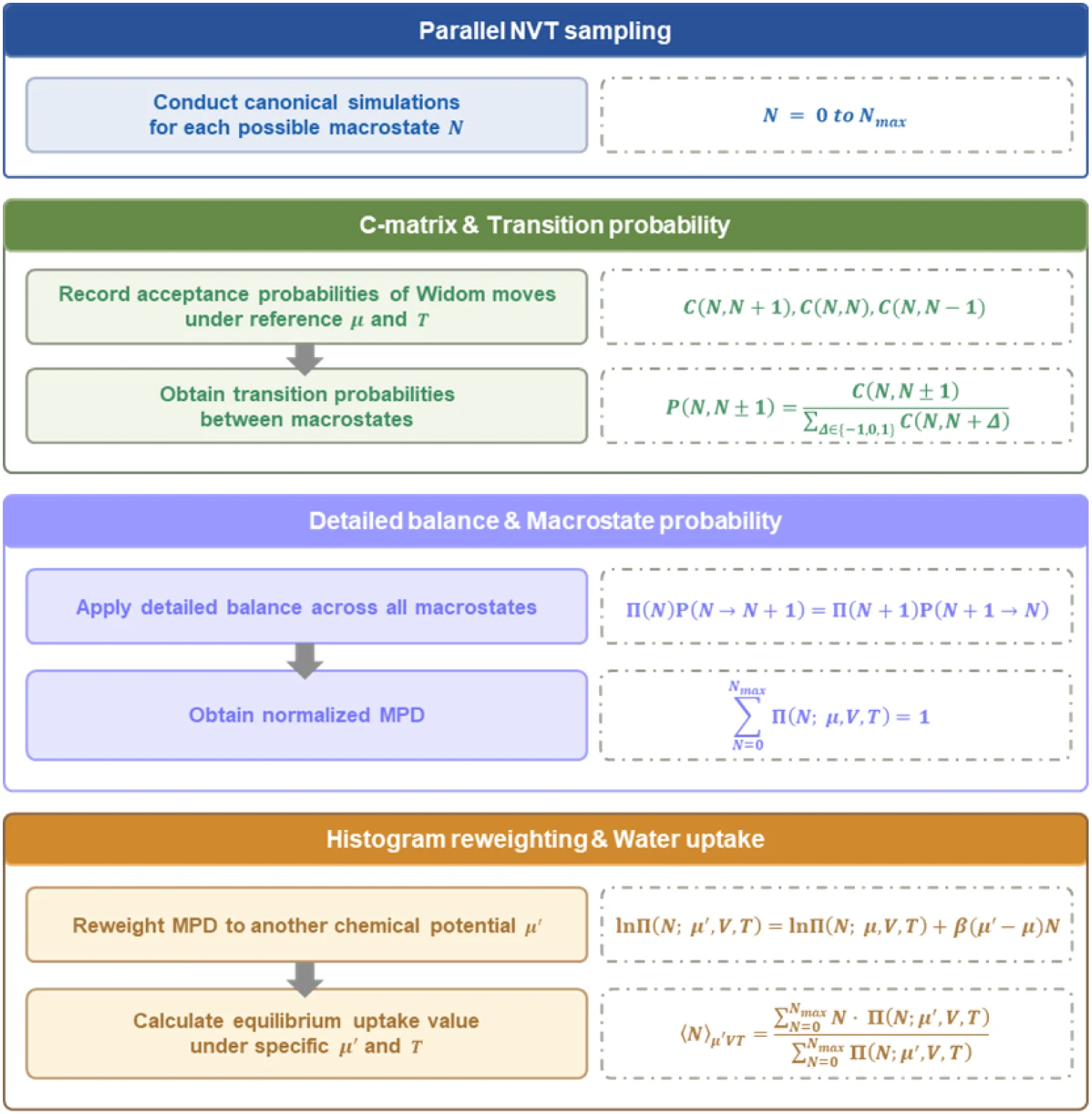
Schematic flowchart of the NVT+W method. The procedure consists of four main stages: (i) Parallel NVT sampling: canonical simulations are conducted for each possible macrostate *N*. (ii) C-matrix and transition probability: the acceptance probabilities of Widom moves are recorded under a reference chemical potential *μ* and temperature *T*, and the transition probabilities between macrostates are obtained. (iii) Detailed balance and macrostate probability: detailed balance is applied across all macrostates to obtain the normalized macrostate probability distribution (MPD). (iv) Histogram reweighting and water uptake: the MPD is reweighted to another chemical potential *μ*′, allowing the equilibrium uptake to be calculated under specific *μ*′ and *T*.

All NVT+W simulations are carried out using an in-house modified version of the open-source RASPA package.^40^ In these calculations, intermolecular interactions are described using 12–6 Lennard–Jones (LJ) potentials and Coulombic interactions with fixed point charges. The LJ parameters for framework atoms are modeled using the DREIDING force field,^41^ with the exception of zirconium ions whose LJ parameters are adopted from the Universal Force Field (UFF).^42^ Detailed force field parameters can be found in Table S1 of the SI. LJ interactions are truncated and shifted to zero at a cutoff distance of 12 Å. The partial charges assigned to the atoms of the MOF-806 framework, calculated using the semiempirical PM7 method^43^ implemented in MOPAC,^44^ are adopted directly from Datar et al.^31^ For the LUDKOM framework, its partial charges are determined using the multilayer connectivity-based atom contribution (m-CBAC) method.^45^ Long-range electrostatic interactions are computed using the Ewald summation method.^46^ Water molecules are represented by the TIP4P-Ew model,^47^ and the corresponding parameters are listed in Table S2. The Lorentz–Berthelot mixing rule is applied for estimating the LJ parameters between different atom types. The simulation box dimensions are set to be at least twice the cutoff radius in all directions. We note that, following the recommendation by Datar et al.,^32,33^ all the presented isotherms are shown in this study as a function of relative pressure that is normalized using the intrinsic saturation pressure of the employed TIP4P-Ew water model (i.e., 986 Pa at 298 K) rather than the experimental value. It should also be noted that this study adopts a fugacity coefficient of unity; therefore, strictly speaking, the pressure corresponds to fugacity.

At this point, we note that, while it remains of importance to validate the adopted molecular potential, direct comparison between simulated and experimental water adsorption isotherms can be complicated due to various uncontrollable factors such as structural defects in experimentally synthesized MOFs. Defects can significantly modify the local adsorption environment, framework–water interactions, and water-clustering behavior, thereby altering the adsorption step and saturation uptake.^38,48,49^ For instance, Choi et al. investigated missing-linker defects with different densities and spatial configurations in MOF-801,^38^ while Chen et al. considered possible metal-node-missing defects in MIL-100­(Fe).^49^ These studies illustrate that the type and density of defects, as well as their spatial distribution can lead to substantially different adsorption behavior. Because the type, density, and spatial distribution of defects in experimental samples are often not fully characterized and are difficult to reproduce comprehensively in simulations, MOF-806 and LUDKOM are treated here as idealized model systems to isolate the fundamental effect of pore connectivity on water adsorption. Nonetheless, although the adopted force fields have not yet been validated against experimental data owing to the difficulties outlined above, the same interaction models are consistently applied to all connectivity variants derived from each parent framework, enabling controlled comparison of relative trends and underlying adsorption mechanisms. Future studies could integrate reference energies computed by density functional theory (DFT) to reparameterize force fields^50–52^ or develop machine learning interatomic potentials (MLIPs)^53,54^ to more accurately describe framework–water interactions. Though, the relative trends observed in this study are expected to remain largely unchanged.

### 2.3 Grand Canonical Monte Carlo Simulations

To compare the adsorption characteristics of water with those of commonly studied nonpolar small molecules, adsorption isotherms of methane, carbon dioxide, and nitrogen at 298 K are also computed. For these adsorbates, conventional GCMC simulations are employed instead of the NVT+W method. The simulations are performed using RASPA with the same force field settings as those described above, while methane, carbon dioxide, and nitrogen are modeled using the TraPPE force field.^55,56^ Translation, rotation, reinsertion, and swap trial moves are attempted with a relative probability of 1:1:2:4, and millions of Monte Carlo cycles are performed until statistically converged equilibrium uptakes are obtained.

In this study, methane is also employed as a nonpolar probe to independently validate the NVT+W implementation, particularly the treatment of blocking spheres. The adsorption of methane is primarily governed by framework–adsorbate interactions and is not expected to exhibit pronounced cooperative condensation at room temperature, allowing conventional GCMC simulations to serve as a suitable reference. As shown in Figure S2a, the two methods yield essentially identical adsorption isotherms for the full LUDKOM framework and all four blocked systems, demonstrating that the NVT+W method consistently reproduces the GCMC results and that the blocking treatment does not introduce artificial bias. Furthermore, the methane insertion transition probabilities at zero loading scale proportionally with the corresponding accessible pore volumes, as shown in Figure S2b, confirming that the decrease in transition probability upon pore blocking primarily reflects the expected reduction in accessible space.

### 2.4 Thermodynamic and Structural Analysis

The grand potential free energy *W* ^57^ for water adsorption in MOFs is analyzed from the obtained MPD (i.e., Π­(*N*; *μ*, *V*, *T*)) as expressed below in eq 1.

1 $$W\left(N;\mu ,V,T\right)=-k_{B}T\;\ln\Pi \left(N;\mu ,V,T\right)$$

In this equation, *k*_B_ is the Boltzmann constant. This free energy profile reveals the relative favorability of different states. For a system undergoing a first-order phase transition from gas phase to a condensed one, the free energy exhibits a double-well shape.^27^ The two wells correspond to the metastable and stable equilibrium states, separated by a free-energy barrier *W*_b_, as illustrated in eq 2. This barrier height can be quantified as

2 $$W_{b}\left(\mu ,V,T\right)=-k_{B}T\;\ln\frac{\Pi \left(N_{local,max};\mu ,V,T\right)}{\Pi \left(N_{local,min};\mu ,V,T\right)}$$

Here, Π­(*N_local_*,*_min_*; *μ*, *V*, *T*) and Π­(*N_local_*,_max_; *μ*, *V*, *T*) represent the macrostate probabilities at the metastable minimum and the local energy maximum, respectively. Conversely, a continuous transition without phase separation simply presents a single-well profile.

The hydrogen-bonding networks of water molecules adsorbed within the MOFs are also analyzed to probe their local environments. Following the geometric criteria of Luzar et al.,^58^ a hydrogen bond is defined by an intermolecular oxygen–oxygen distance of less than 3.5 Å and an O–H···O angle smaller than 30°. As illustrated in Figure 3a, each water molecule can in principle form up to four hydrogen bonds by acting as both a donor and an acceptor. Since the number of confined molecules varies across different structures and conditions, this analysis is performed using the loading N with the highest probability at the saturation pressure (i.e., *P*/*Psat* = 1). Furthermore, the local environment of each adsorbed water molecule is further classified into three categories depending on its number of hydrogen-bond donors: double-donor (DD), single-donor (SD), and none-donor (ND), as schematically depicted in Figure 3b. A DD molecule donates two hydrogen bonds through both of its hydrogen atoms and an SD molecule donates only one hydrogen bond, while an ND molecule does not donate any hydrogen bonds and may only act as an acceptor. The relative population (i.e., fraction) of DD, SD, and ND is subsequently quantified, serving as a statistical descriptor of the hydrogen-bonding environment.^59^

**Figure 3:**
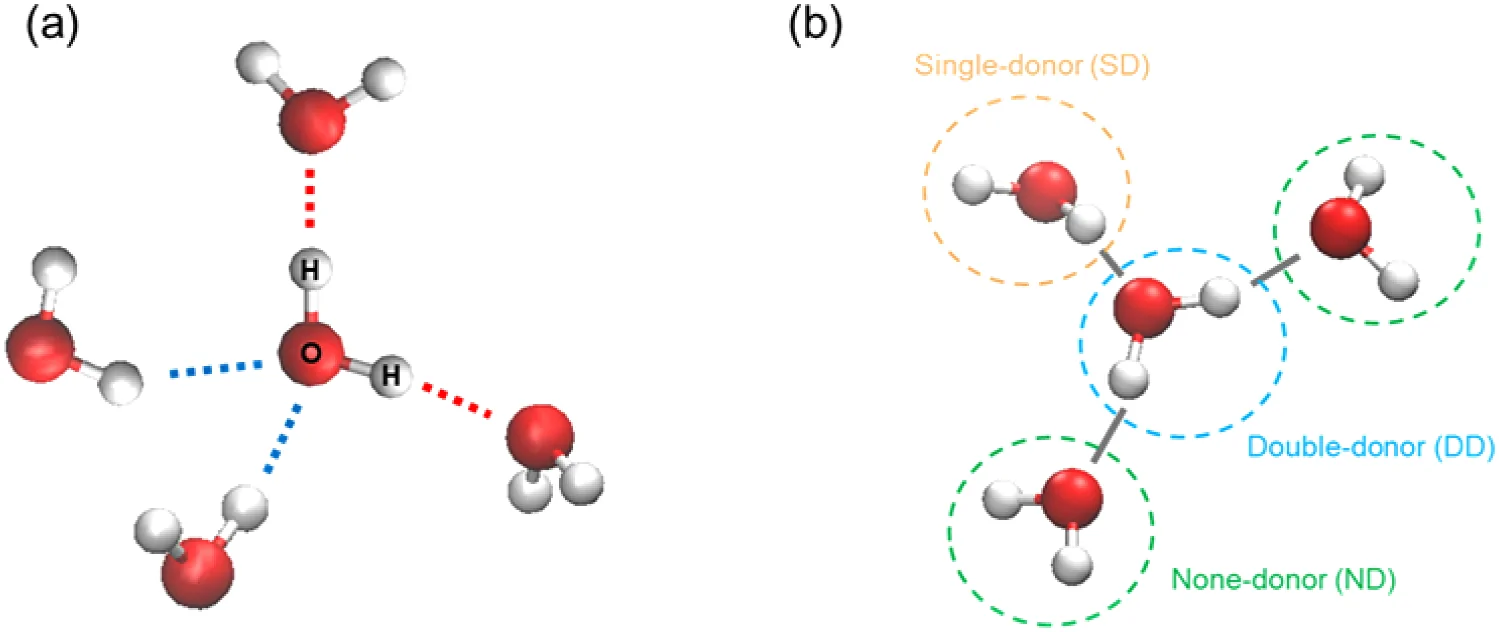
Schematic illustration of (a) hydrogen-bond network surrounding a water molecule that can form up to four hydrogen bonds, including two donors and two acceptors, and (b) specific hydrogen-bond donor configurations: double-donor (DD), single-donor (SD), and none-donor (ND).

Besides, given the strongly associating nature of water molecules, their adsorbate–adsorbate (AA) interactions are quantified in this study through an energy decomposition approach based on molecular configurations at saturated loading. Specifically, the total AA interaction energy of the whole system is first evaluated. Subsequently, a water molecule is removed from the configuration, and the AA interaction energy of the remaining system is recalculated. The difference between these two energy values is the contribution of the removed molecule to the overall AA interaction energy. Note that a factor of 50% is applied to account for the double counting of pairwise interactions. This procedure is repeated for each water molecule in the system, yielding molecule-specific AA interaction energies. With the interaction energy for each water molecule and its location at our disposal, analyses are then carried out to obtain two-dimensional energy maps. Specifically, the molecular positions are projected onto the cross-sectional plane that is perpendicular to the channel direction, after which the interaction energies associated with molecules located within each meshed bin are averaged to generate a two-dimensional energy map. To ensure reasonable statistics, 10,000 equilibrium configurations are extracted from NVT simulations at the saturation loading obtained at the relative pressure of a unity (i.e., P/*Psat* = 1).

## 3 Results/Discussion

Herein, Section 3.1 first examines highly interconnected MOF-806 frameworks by analyzing their adsorption isotherms, thermodynamic stability, and microscopic water structures. This analysis clarifies how extended spatial connectivity promotes cooperative hydrogen bond network formation, stabilizes the high-density adsorbed phase, and shifts the adsorption step toward lower pressure. Section 3.2 focuses on LUDKOM, which consists of independent one-dimensional channels, and the results show that the adsorption in different channels can still be coupled through long-range AA interactions. Finally, Section 3.3 compares the adsorption of water with that of nonpolar gases, demonstrating that pore connectivity has a unique effect on the former.

### 3.1 MOFs of Interconnected Cages

#### 3.1.1 MOF-806

Figure 4a first shows the adsorption isotherm of water in MOF-806 with fully connected cages, which exhibits a sharp step at a relative pressure of 0.683. At low pressures, only a limited amount of water is adsorbed, indicating weak initial interactions between water molecules and the framework. Once the relative pressure approaches 0.683, the water uptake increases rapidly, demonstrating the characteristic S-shaped adsorption behavior. Correspondingly, the MPD of water adsorption in the fully connected MOF-806 depicted in Figure S3a exhibits bimodal behavior at this relative pressure, indicating a first-order phase transition upon adsorption. The adsorption behavior of individual pores is also examined using blocked frameworks containing only isolated large or small pores. As shown in Figure 4a, both the isolated large and small pores similarly exhibit S-shaped isotherms with a sharp adsorption step. Their MPDs also demonstrate bimodal distributions near the corresponding step pressures, as can be respectively seen from Figure S3b and S3c, confirming that adsorption in the isolated pores again proceeds through first-order phase transitions. Notably, the adsorption steps of the isolated pores are interestingly found to shift to significantly higher pressures relative to that of the fully connected framework. This comparison indicates that, while individual cages can still exhibit sharp S-shaped adsorption behavior, the connection of cages substantially lowers the pressure at which the adsorption transition occurs. Note that the isolated small-pore system includes all eight small pores within the simulation cell as noted above. An additional comparison between an isolated large pore and a system containing only one accessible small pore is shown in Figure S4 of the SI with related discussion.

**Figure 4:**
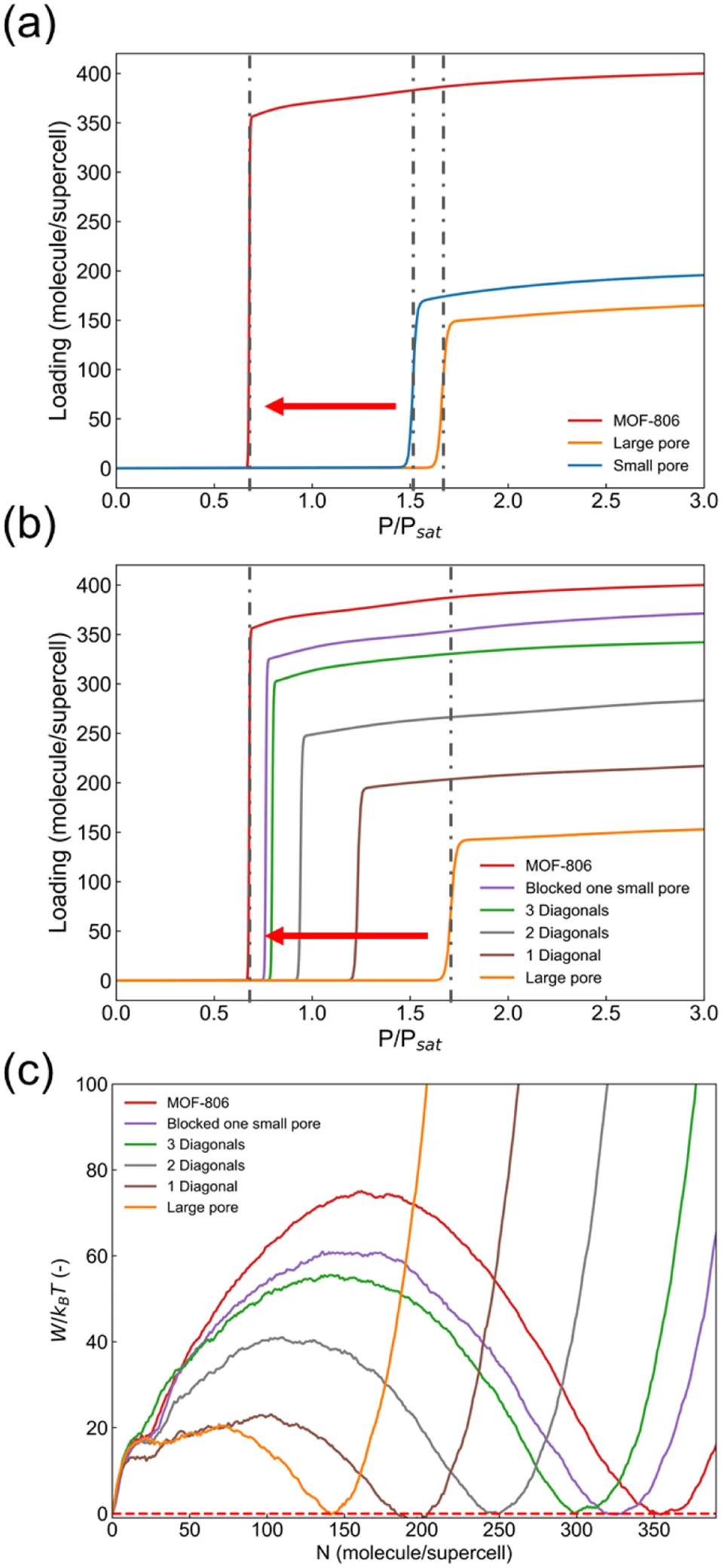
(a) Simulated water adsorption isotherms in MOF-806 and that of its individual cage (i.e., large or small). Vertical dashed lines indicate their respective step pressures. (b) Water adsorption isotherms in a series of MOF-806-based structures with varying degrees of pore connectivity, ranging from an isolated large pore to the fully connected network. (c) Grand potential free energy profiles evaluated at the respective step pressures for the model structures shown in (b).

This connectivity effect is further investigated using a series of model structures with varying levels of pore connectivity. As introduced above, these models range from an isolated large pore to a fully connected pore network, with intermediate configurations including structures containing one, two, and three diagonal connections, as well as a structure with one blocked small pore. The resulting NVT+W isotherms, shown in Figure 4b, further demonstrate the critical role of structural connectivity in governing water adsorption behavior. As the pore connectivity increases, the adsorption step progressively shifts toward lower pressures.

To gain further insight into the thermodynamic origin of this behavior, the grand potential free energy *W* as a function of water uptake is analyzed at the corresponding step pressures, with the results summarized in Figure 4c. In all cases, two free-energy minima are observed, corresponding to the low-density and high-density adsorbed phases, and these minima are separated by a free-energy barrier. With increasing pore connectivity, the free-energy barrier becomes notably greater, suggesting a more pronounced difference between the gas and the condensed phase. This also implies that the condensed phase formed in MOF is more bulk-like, which is further confirmed from microscopic analyses as will be detailed later, and is more stable. Indeed, if the free-energy profiles are analyzed at the same relative pressures, as shown in Figure S5, the systems with higher pore connectivity exhibit lower free energies in the high-loading region relative to the low-loading state. This result confirms that greater pore connectivity stabilizes the condensed water phase.

To shed some light on the more bulk-like phase in frameworks of connected cages as discussed above, the hydrogen-bond network formed between adsorbed water molecules is analyzed. Notably, Figure 5a shows that the average number of hydrogen bonds decreases as pore connectivity is reduced, from 3.28 in the fully accessible MOF-806 structure to 3.00 in the isolated large pore. This trend indicates that water molecules form a less extended hydrogen-bond network in more isolated pore environments. The hydrogen-bond donor configurations, including DD, SD, and ND states, are also examined with results shown in Figure 5b. The fully connected MOF-806 exhibits a donor-state distribution closer to that of bulk water, with a higher DD ratio of nearly 70%. Reducing pore connectivity shifts the distribution toward significantly lower donor coordination (i.e., ∼55% in a single large cage). To further identify the spatial origin of this connectivity-dependent trend, the average number of hydrogen bonds per water molecule and the distributions of DD, SD, ND water molecules are computed as a function of distance from the pore center within the common large pore. The large pore is selected because it is retained in all MOF-806 variants, allowing comparison within an equivalent pore environment. As shown in Figure S6, the most pronounced differences among the connectivity variants occur near the connections between the large pore and the neighboring small pores. In this region, the average number of hydrogen bonds decreases progressively as pore connectivity is reduced. Correspondingly, this decrease is mainly associated with a reduction in the DD fraction, accompanied by increases in the SD and ND fractions. Collectively, these results suggest that pore connectivity facilitates the formation of a more complete hydrogen-bond network among confined water molecules. Highly connected cages in MOFs allow the hydrogen-bond network to extend across neighboring cavities, thereby stabilizing the high-density adsorbed phase and thus enabling the transition to occur at a lower chemical potential value (i.e., lower step pressure).

**Figure 5:**
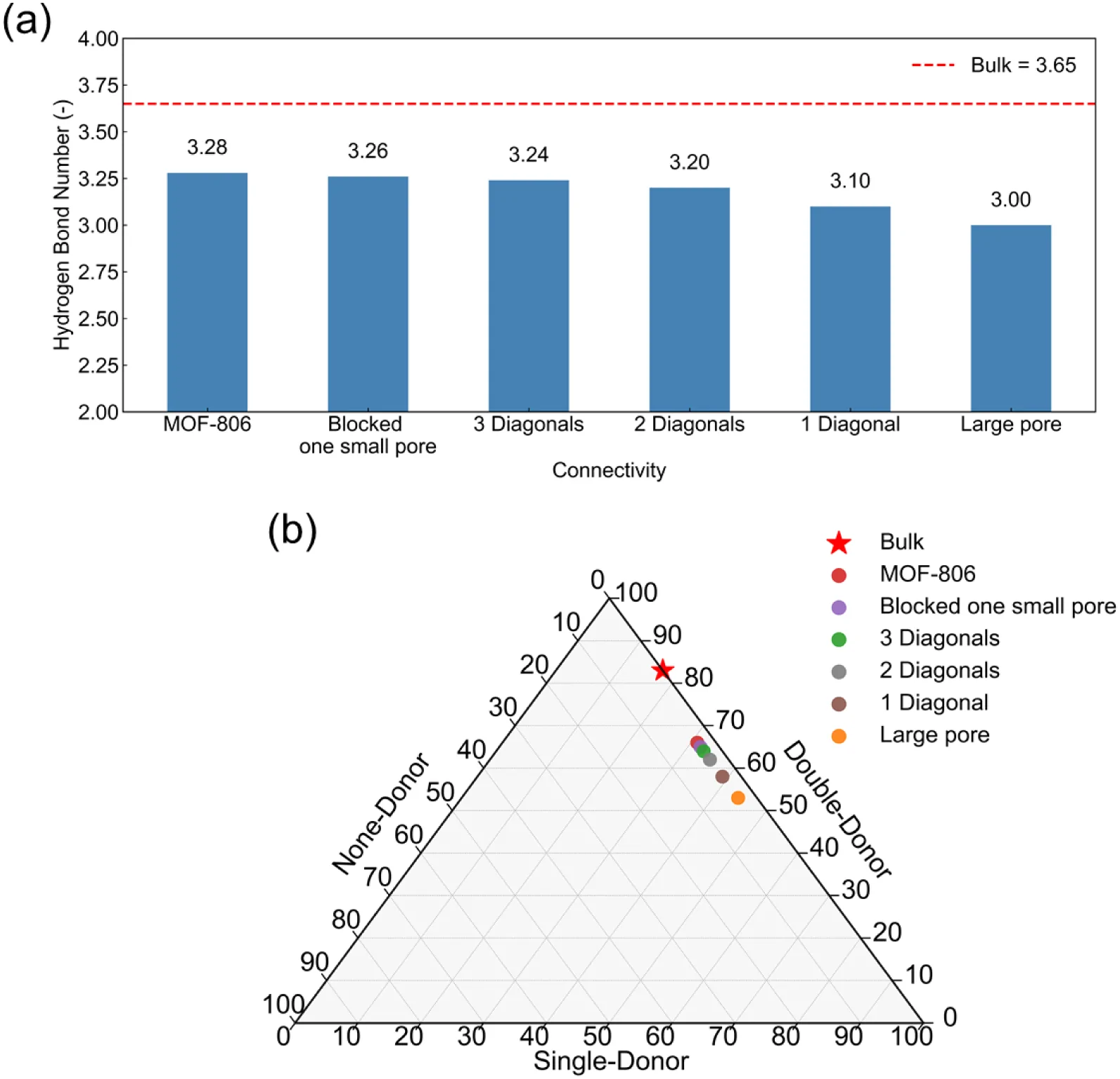
Hydrogen-bond network analysis of adsorbed water molecules in MOF-806 with varying pore connectivity. (a) Average number of hydrogen bonds per water molecule. (b) Ternary plot for the distribution of different hydrogen-bond donor configurations (i.e., double-donor, single-donor, and none-donor).

### 3.2 MOFs of Independent Channels

#### 3.2.1 LUDKOM

LUDKOM is then examined as an extreme case of low pore connectivity to clarify how communication between distinct pore domains influences water adsorption. Unlike MOF-806, in which neighboring cages are directly connected, LUDKOM contains two types of spatially independent one-dimensional channels without direct molecular connection between them. This framework therefore enables us to determine whether water adsorption in one channel can affect adsorption in another even when the channels are not geometrically connected.

The adsorption isotherm of water in LUDKOM (Figure 6a) exhibits a distinct stepwise behavior, where significant water uptake first occurs in the large channels at low pressures, followed by a sharp second adsorption step in the small channels at relatively higher pressures. This sequential filling behavior can be more clearly seen from the MPD shown in Figure S3d–f. The large-channel system exhibits a single prominent peak (i.e., unimodal distribution) at high loading under a lower pressure, whereas the small-channel system shows a bimodal distribution, indicating that adsorption in the small channels likewise proceeds through a first-order transition but occurs at a higher pressure. This channel-dependent adsorption sequence is consistent with the stronger host–adsorbate interaction in the large channels (i.e., 37.22 vs 29.92 kJ/mol). Water preferentially fills the more energetically favorable large channels at low pressures before adsorption occurs in the smaller ones at higher pressures.

**Figure 6:**
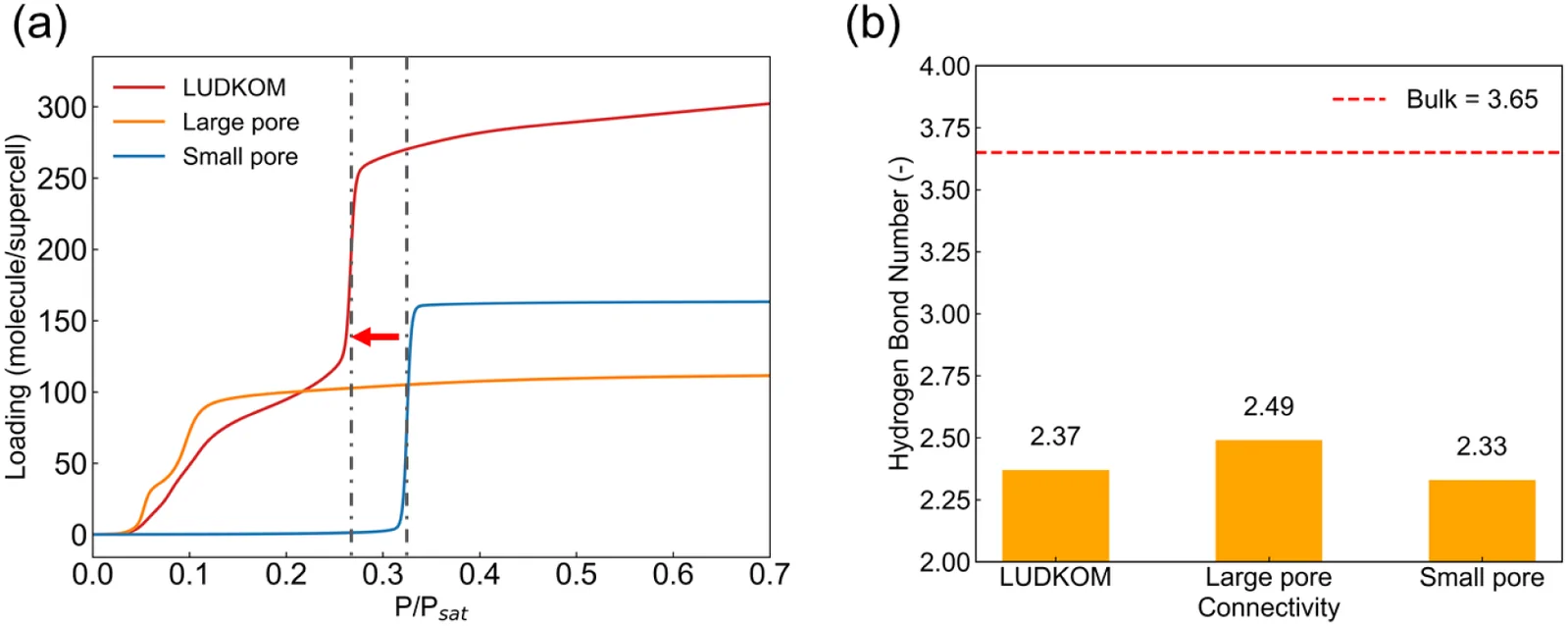
Water adsorption behavior and hydrogen bond number in the low-connectivity LUDKOM framework. (a) Simulated adsorption isotherms of water in the complete LUDKOM structure and its isolated large or small channels. Vertical dashed lines indicate their respective step pressures. (b) Average number of hydrogen bonds per water molecule for the complete LUDKOM structure and each of its individual channels. The red dashed line denotes the reference value for bulk water.

Notably, despite the structurally independent nature of these channels (i.e., no direct connection), the adsorption behavior within individual channels is still not completely isolated from that of the entire framework. Specifically, the adsorption step associated with the small channels (i.e., the second step featuring a first-order transition) shifts slightly toward lower pressures once the neighboring large channels become occupied. This suggests that adsorbed water within the large channels can still exert a stabilizing influence on nearby channels, although the effect remains much weaker than the cooperative condensation observed in the highly interconnected MOF-806 as discussed above. A similar trend is also observed for WOJJOV, which likewise contains isolated one-dimensional channels^60^ (Figure S7), further supporting that weak interpore influence can persist even in systems with highly limited connectivity. More details on the structure and related results can be found in the SI.

To understand the origin of this observed shift, the role of intermolecular hydrogen bonding is first examined, following the analysis conducted above for MOF-806. Specifically, the average number of hydrogen bonds for water adsorbed in LUDKOM and in each individual channel is computed. As shown in Figure 6b, the overall framework exhibits an average of 2.37 hydrogen bonds per water molecule, which is close to the averaged behavior of the individual channel systems. More precisely, the average hydrogen-bond number in the whole LUDKOM framework is even marginally lower than the average of that in each independent channel. The results suggest that water molecules in the full framework experience hydrogen-bonding environments similar to those in the isolated channels, indicating that the coexistence of adsorbed water in distinct channels does not promote cross-pore hydrogen-bond connectivity. This appears reasonable, provided that the physical distance between independent channels is greater than the cutoff distance of hydrogen bonds. Nonetheless, the slight shift in step pressure cannot be primarily attributed to strengthened hydrogen bonding, implying that other intermolecular interactions are responsible for the remaining differences.

Given that water possesses a permanent dipole moment with significant long-range electrostatic interactions, water molecules may still influence one another even when the channels within the structure are geometrically independent. To probe this possibility, the spatial distribution of the interaction energies between adsorbates (i.e., AA interactions) is further examined. As shown in Figure 7a, the AA interaction map of the fully adsorbed system reveals that the most energetically stabilized water molecules are primarily located near the channel centers rather than adjacent to the framework surface. That is, water molecules located closer to the channel surface in fact exhibit weaker AA stabilization, likely because they have fewer neighboring water molecules within the confined geometry. Nevertheless, this weaker AA stabilization near the channel periphery does not necessarily exclude contributions from water molecules in adjacent channels.

**Figure 7:**
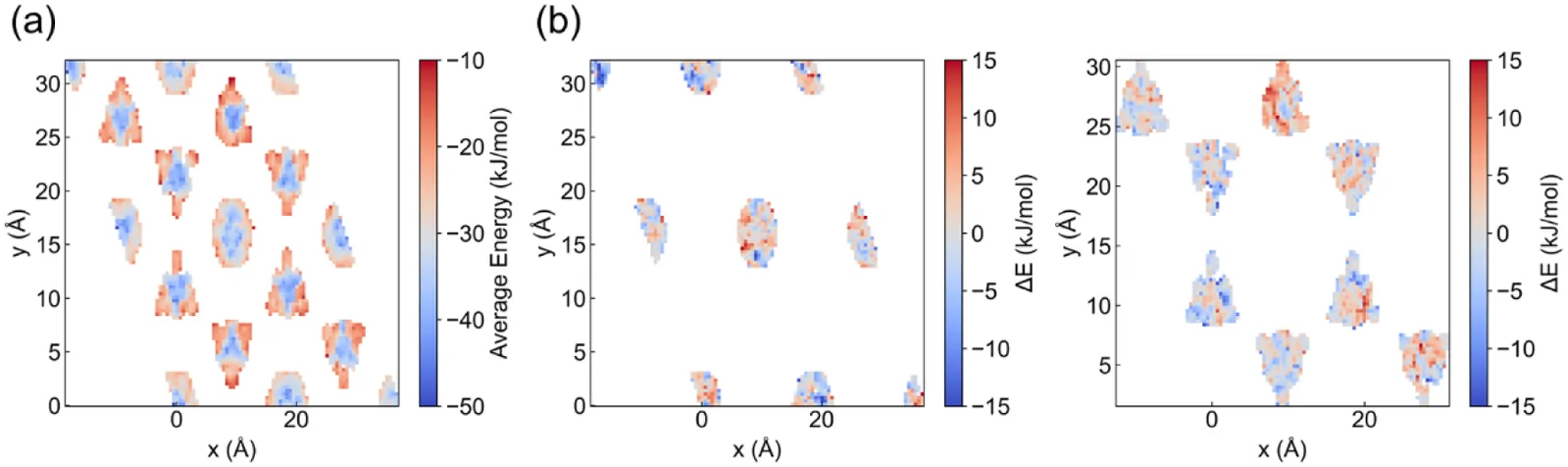
(a) Spatial AA energy distribution of water molecules adsorbed in the LUDKOM framework. (b) Energy-difference (Δ*E*) maps obtained by subtracting the AA interactions of water molecules residing in the isolated large channels and small channels from those in the whole system (i.e., shown respectively in the left and right). The negative values near the channel boundaries indicate additional stabilization derived from cross-pore interactions.

Herein, the AA interaction energies in the full LUDKOM structure are further compared with those obtained from the isolated channel systems. Figure 7b presents maps of the AA interaction energy difference Δ*E*, obtained by subtracting the AA interaction energies of the corresponding isolated channel systems from those of the full LUDKOM system. The energy-difference maps interestingly reveal that localized regions near the channel boundaries appear to exhibit slightly more favorable AA energies in the full framework relative to the isolated channel models. This suggests that water molecules in adjacent channels can indeed still influence one another through long-range electrostatic interactions, thereby facilitating the stabilization of adsorption.

### 3.3 Distinct Adsorption Behaviors between Nonpolar Molecules and Water

To highlight the uniqueness of water adsorption on pore connectivity, we also compute the adsorption isotherms of nonpolar molecules, specifically methane, carbon dioxide, and nitrogen, in the same series of MOF systems. As is known, the adsorption behavior of these small molecules is predominantly governed by host–adsorbate interactions.^61^ Consequently, their isotherms typically exhibit non-S-shaped, Langmuir-like profiles. Therefore, unlike water adsorption, modifying pore connectivity is expected to have only a limited influence on the adsorption behavior, apart from the direct change in accessible void volume. That is, upon restricting pore connectivity, the adsorption of these molecules will likely remain in the same shape but with reduced maximum capacity per their accessible volume. Indeed, as shown in Figure 8, blocking specific regions proportionally reduces the maximum adsorption capacity for all three adsorbates, while the overall isotherm shape remains the same without any noticeable shift in adsorption pressure. To better quantify this behavior, the relative difference between the uptake in each studied system and the corresponding uptake sum of all individual channels/cages is computed with results shown in Figure 8. As an example, for the “1 Diagonal MOF-806” framework consisting of a central large cage and two surrounding cages, its GCMC-computed uptake is compared with the uptake sum of one individual large cage and two small cages. As shown in Figure 8a–c for the adsorption of the three gases in MOF-806-based systems, the difference is nearly zero though some minor deviations are present. Such deviation stems from the fact that each individual cage in such a highly connected structure cannot be perfectly divided. As such, minor overlaps or small omitted regions occur upon blocking and thus lead to the observed differences. Nevertheless, these deviations can be deemed marginal. Similarly, for LUDKOM with spatially independent channels, Figure 8d–f shows that, as would be anticipated, the summed uptake of the individual channels resembles that of the entire framework. Overall, these results validate that, for nonpolar adsorbates as studied herein, adsorption can be largely interpreted as additive contributions from the accessible pore regions. By contrast, as demonstrated in the previous sections, water adsorption is instead strongly influenced by the pore connectivity; changes in the pore connectivity may not only affect the total water uptake but also induce a clear shift in the adsorption step pressure.

**Figure 8:**
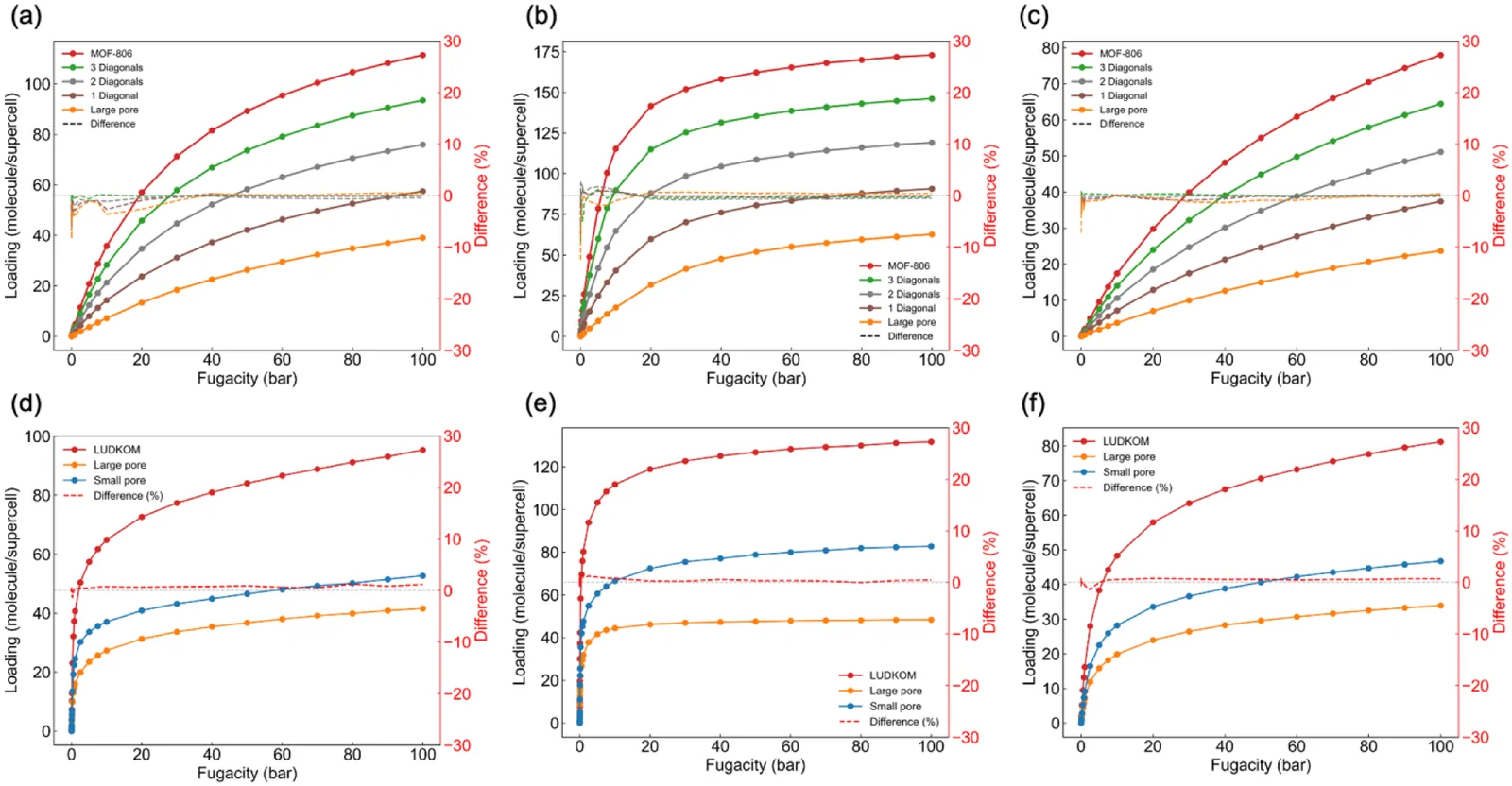
Adsorption uptake (left axis) of (a, d) methane, (b, e) CO_2_, and (c, f) N_2_ in (a−c) MOF-806-based and (d−f) LUDKOM-based systems. The corresponding relative difference between the simulated uptake and the sum of the contributions from individual cages/channels is shown on the right axis.

## 4 Conclusions

Through investigating representative structures of distinct topologies coupled with strategic blocking using flat-histogram Monte Carlo (NVT+W), this study demonstrates the key role of pore connectivity in controlling water adsorption in MOFs. From the study of MOF-806 with highly connected cages, our results clearly reveal that increased connectivity can enable water adsorption with a more complete hydrogen-bond network. This promotes cooperative pore filling, enabling its occurrence at a lower relative pressure or smaller chemical potential value. Notably, even for a MOF comprising fully independent channels where hydrogen bonds cannot be formed between water molecules residing in different channels, long-range Coulombic interactions between water molecules can still influence their adsorption behavior to a certain extent. As shown by the spatial energy analysis, the enhanced AA contribution for water molecules near the channel surface offers additional stabilization to their adsorption in the full framework compared to that in each individual channel. That is, this energetic coupling between neighboring pore regions lowers the adsorption step pressure, indicating that water adsorption can be influenced not only by direct pore connectivity but also by adsorbate–adsorbate stabilization across spatially adjacent pores. Finally, we have also shown that the influence of pore connectivity is unique to strongly associated molecules. Overall, this work advances our understanding of water adsorption in MOFs, particularly the effect of pore connectivity, offering valuable insights for the rational design of efficient water-harvesting adsorbents.

## Supplementary Material



## Notes

Additional details and data referred to in the main text, including pore size distribution (PSD) analyses, details of NVT+W calculations, validation of the pore-blocking approach implemented in NVT+W calculations, force field parameters, MPD profiles of water adsorption in MOF-806 and LUDKOM, comparison of water adsorption and hydrogen-bond formation in the large pore and one small pore of MOF-806, grand potential free energy profiles of water in MOF-806 at selected relative pressures, spatial hydrogen-bonding analyses, and water adsorption in WOJJOV (i.e., another low-connectivity MOF studied herein) (PDF)
