# Supplementary material for: Effects of Pore Connectivity on Water Adsorption in Metal–Organic Frameworks
Source: Langmuir. 2026 Aug 11;42(33):24443–54. doi: 10.1021/acs.langmuir.6c03373 (PMC13523753; doi:10.1021/acs.langmuir.6c03373)
Supplement: Supplementary file 1 [file la6c03373_si_001.pdf]

Supporting Information

for

Effects of Pore Connectivity on Water Adsorption in Metal–Organic Frameworks

Yen-Ju Chu[a] *and Li-Chiang Lin*[a,*]

[a]Department of Chemical Engineering, National Taiwan University, No. 1, Sec. 4, Roosevelt Road, Taipei 106319, Taiwan

*Email: lclin@ntu.edu.tw

Table of Contents:

1. Pore size distribution analysis of MOF-806 and LUDKOM
2. NVT+W simulations
3. Force field parameters
4. Validation of the pore-blocking approach implemented in the NVT+W calculations
5. MPD of water adsorption in MOF-806 and LUDKOM
6. Comparison of water adsorption and hydrogen-bond formation in the large pore and one small pore
7. Free energy comparison at given relative pressures
8. Spatial hydrogen-bonding analyses
9. Low-connectivity structure: WOJJOV

1. Pore size distribution analysis of MOF-806 and LUDKOM

The pore size distribution (PSD) of MOF-806 and LUDKOM, calculated using Zeo++ with a probe diameter of 2.65 Å, are presented in **Figure S1**. Because both frameworks contain two primary pore environments, the maxima of the two dominant PSD peaks are used as the representative pore diameters, as these peak positions reflect the characteristic local dimensions of the corresponding pores or channels. As shown in **Figure S1a**, MOF-806 exhibits two dominant peaks at approximately 10.0 and 12.8 Å, which are assigned to the surrounding small pores and the central large pore, respectively. Note that several additional minor peaks are also observed because the large and small pores form a highly interconnected three-dimensional cage network. That is, the local pore dimensions vary near the cage openings and connected regions between neighboring pores, thus giving rise to additional features in the PSD. By contrast, **Figure S1b** shows two sharp PSD peaks at approximately 6.1 and 6.8 Å for LUDKOM, corresponding to its small and large channels, respectively. Because the two channel types are spatially isolated and possess relatively uniform local pore dimensions, additional peaks as would be expected are not observed.

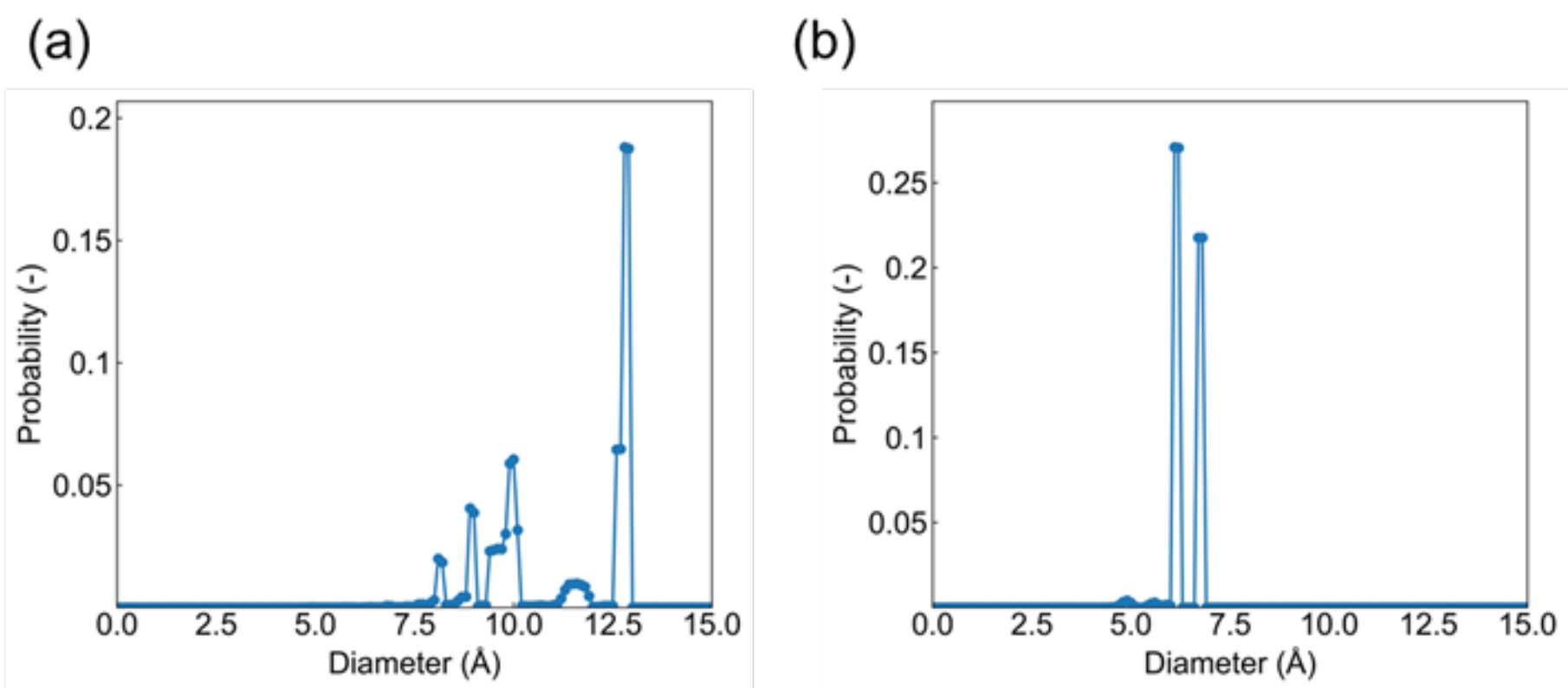

Figure S1. Pore size distributions of (a) MOF-806 and (b) LUDKOM. The dominant peak maxima are used as representative pore diameters. MOF-806 exhibits peaks corresponding to the small and large pores at approximately 10.0 and 12.8 Å, respectively, together with minor features arising from its interconnected cage network.

LUDKOM exhibits two well-defined peaks corresponding to the small and large channels at approximately 6.1 and 6.8 Å, respectively.

2. NVT+W simulations

The NVT+W method[1-4] is a flat-histogram Monte Carlo approach designed to determine the macrostate probability distribution $\Pi(N;\ \mu, V, T)$ under a specified reference chemical potential μ, volume V, and temperature T. Once obtained, the MPD can be used to calculate the equilibrium average loading under any desired conditions. In this method, independent canonical simulations are performed for each possible loading macrostate *N*, ranging from the empty state to the estimated saturation loading ($N = N_{max}$). During each NVT simulation, Widom insertion or deletion moves are carried out to probe the statistical tendency of the system to accept or delete one molecule. Note that these are ghost moves and are never actually accepted. The acceptance probability of each attempted Widom move, per the grand canonical potential under the reference chemical potential and temperature, is accumulated in the C-matrix as follows:

- For insertion moves ($N \rightarrow N + 1$):

$$C(N, N+1) = C(N, N+1) + \alpha(\Gamma^N \rightarrow \Gamma^{N+1})$$

$$C(N, N) = C(N, N) + [1 - \alpha(\Gamma^N \rightarrow \Gamma^{N+1})]$$

$$\alpha(\Gamma^N \rightarrow \Gamma^{N+1}) = \min\,[1, \frac{\beta V \phi P}{N+1} e^{-\beta(U(\Gamma^{N+1}) - U(\Gamma^N))}]$$

- For deletion moves ($N \rightarrow N - 1$)

$$C(N, N-1) = C(N, N-1) + \alpha(\Gamma^N \rightarrow \Gamma^{N-1})$$

$$C(N, N) = C(N, N) + [1 - \alpha(\Gamma^N \rightarrow \Gamma^{N-1})]$$

$$\alpha(\Gamma^N \rightarrow \Gamma^{N-1}) = \min\,[1, \frac{N}{\beta V \phi P} e^{-\beta(U(\Gamma^{N-1}) - U(\Gamma^N))}]$$

where $C(N, N \pm 1)$ represents the accumulated acceptance probabilities associated with Widom insertion ($(N, N+1)$) and deletion moves ($(N, N-1)$), while $C(N, N)$ accounts for the corresponding rejection probabilities of both move types. The term α denotes the acceptance probability, and Γ^N, Γ^{N+1}, and Γ^{N-1} respectively correspond

to a system configuration at macrostates N, $N+1$, and $N-1$. β is equal to $(k_B T)^{-1}$, where k_B is the Boltzmann constant. In the acceptance criteria, ϕ and P denote the fugacity coefficient and reference pressure, respectively, such that ϕP corresponds to the reference fugacity at which the C-matrix elements are accumulated. Herein, the fugacity coefficient is set to unity such that this pressure considered is numerically equal to the fugacity.

Transition probabilities and macrostate probability distribution: Once the C-matrix statistics have converged through monitoring the so-called C-ratio ($C(N, N-1)/C(N, N+1)$) (i.e., the ratio remains approximately a constant), the unbiased transition probabilities $P(N, N \pm 1)$ can then be extracted from the matrix elements as shown below:

$$P(N, N+\Delta) = \frac{C(N, N+\Delta)}{\sum_{\Delta \in \{-1,0,1\}} C(N, N+\Delta)}$$

where $P(N \rightarrow N+\Delta)$ is the probability of the system from macrostate N to $N+\Delta$, with $\Delta = -1, 0, 1$.

These probabilities are then linked to the macrostate probability distribution (denoted as MPD), $\Pi(N)$, through the condition of detailed balance:

$$\Pi(N)P(N \rightarrow N+1) = \Pi(N+1)P(N+1 \rightarrow N)$$

By iteratively applying this relation for each value of N, the relative probability across all macrostates can be constructed starting from a reference state (e.g., $N=0$). After proper normalization, the MPD under the given reference state can then be obtained.

Histogram reweighting and isotherm loading: A significant advantage of this framework is the ability to predict thermodynamic observables over a continuous range

of conditions via histogram reweighting. For this study to obtain the adsorption isotherm under a given temperature, the MPD obtained at a reference chemical potential μ can be reweighted to a new chemical potential μ' at a constant temperature T using the following relation:

$$\ln\Pi(N;\ \mu', V, T) = \ln\Pi(N;\ \mu, V, T) + \beta(\mu' - \mu)N$$

With these, the MPD under any pressure or fugacity values can be obtained; the corresponding equilibrium uptake $\langle N \rangle$ is simply the weighted average of all macrostates:

$$\langle N \rangle_{\mu' VT} = \frac{\sum_{N=0}^{N_{max}} N \cdot \Pi(N;\ \mu', V, T)}{\sum_{N=0}^{N_{max}} \Pi(N;\ \mu', V, T)}$$

3. Force field parameters

The Lennard–Jones (LJ) parameters for framework atoms (**Table S1**) are adopted from the DREIDING[5] force field, while Zr and Cu atoms are described using the UFF force field[6]. Water molecules are modeled using the TIP4P-Ew[7] model (**Table S2**).

Table S1. LJ parameters for framework atoms

Atom Type	ε/k_B (K)	σ (Å)
Zr	34.72	2.78
Cu	2.52	3.11
Al	156.00	3.91
C	47.86	3.47
H	7.65	2.85
O	48.18	3.03

Table S2. Parameters of the adopted water TIP4P-Ew molecular model

Atom Type	ε/k_B (K)	σ (Å)	q (-)
O	81.92	3.16	0.000
H	0.00	0.00	0.524
M	0.00	0.00	-1.048

4. Validation of the pore-blocking approach implemented in the NVT+W calculations

To validate the implementation of blocking spheres in the NVT+W simulations, methane adsorption isotherms are computed using both GCMC and NVT+W simulations. Methane is selected as a nonpolar probe molecule because its adsorption is mainly governed by host–adsorbate interactions, and strong cooperative adsorption behavior is not expected. Therefore, conventional GCMC simulations provide a reliable reference for evaluating the NVT+W results. As shown in **Figure S2a**, the two methods give nearly identical adsorption isotherms for the full LUDKOM framework and the four blocked systems, including the large-channel-only, small-channel-only, one-large-channel, and one-small-channel systems. This agreement confirms that the pore-blocking treatment does not introduce artificial effects into the NVT+W isotherm calculations.

In addition to the isotherm comparison, the relationship between pore blocking and transition probability is analyzed. Because methane trial moves that fall inside the blocking pockets are rejected, pore blocking may reduce the insertion transition probability included in the C-matrix calculation. To examine whether this decrease is mainly associated with the reduction in accessible pore volume, the normalized transition probability for methane insertion, $P(N \rightarrow N+1)$, is compared with the normalized accessible pore volume at ($N = 0$). As shown in **Figure S2b**, the blocked LUDKOM systems closely follow the ideal proportional relationship between accessible pore volume and insertion transition probability.

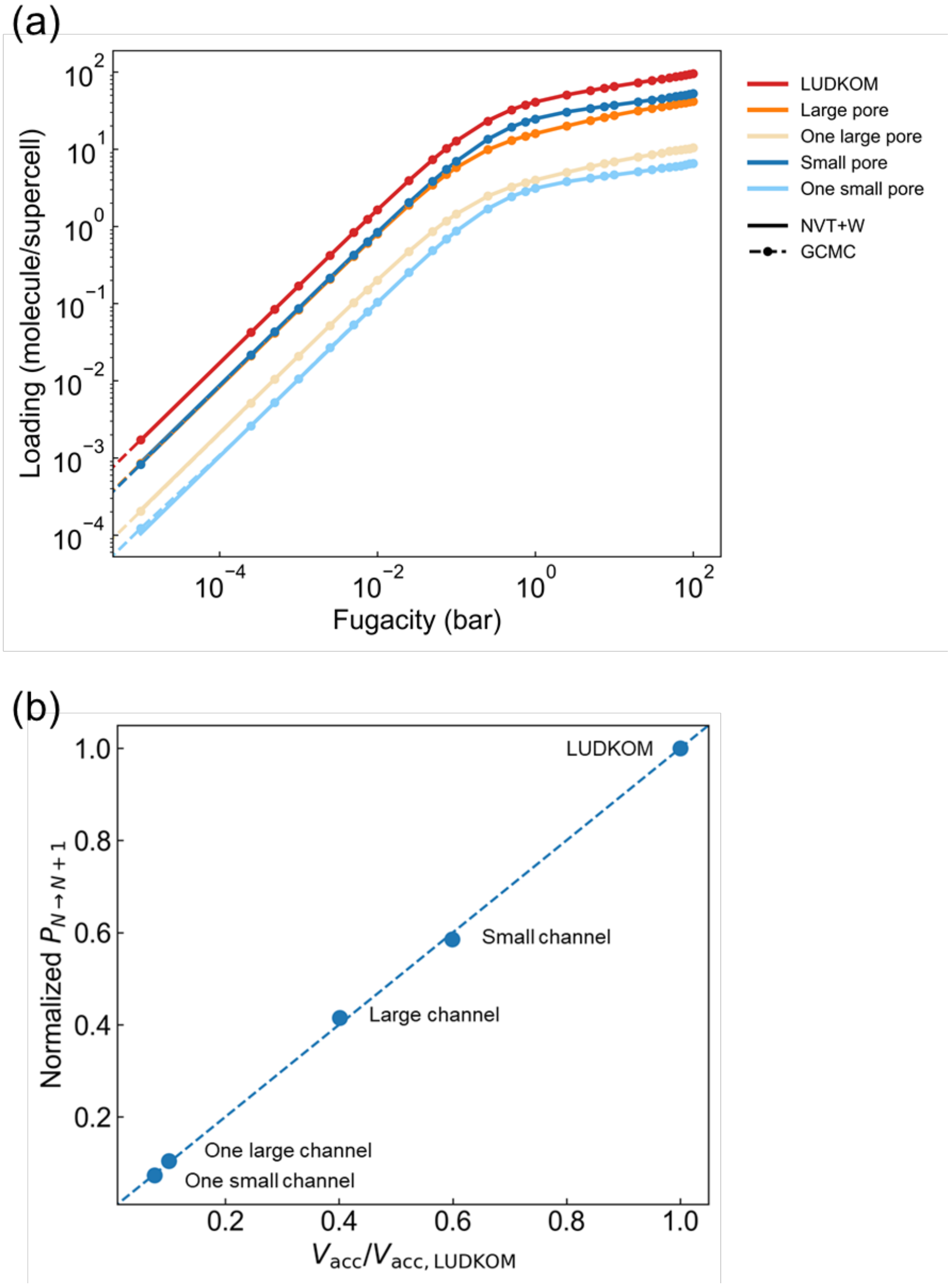

Figure S2. (a) Methane adsorption isotherms in the entire LUDKOM structure and in the modified structures with only large channels or only small channels. The excellent agreement between the two methods validates the implementation of pore blocking in the NVT+W calculations. (b) Relationship between the normalized accessible pore volume and the normalized transition probability for methane insertion from N to $N+1$ in LUDKOM-based systems. Both the accessible pore volume and transition probability are normalized by the corresponding values of the full LUDKOM structure. The dashed line represents a linear proportional relationship. The close agreement

between the data points and the dashed line indicates that the decrease in transition probability mainly follows the reduction in accessible pore volume caused by pore blocking.

5. MPD of water adsorption in MOF-806 and LUDKOM

The MPD profiles corresponding to the adsorption of MOF-806 and LUDKOM discussed in the main text are summarized in **Figure S3**. **Figure S3a–c** show the MPD for MOF-806, including the fully connected framework, the isolated large-pore system, and the isolated small-pore system, respectively. These MPD profiles exhibit bimodal distributions near their corresponding step pressures, indicating the coexistence of low- and high-loading states. This confirms that the sharp adsorption steps observed in both the fully connected MOF-806 framework and the isolated pore systems are associated with first-order phase transitions.

For LUDKOM, the MPD profiles of the full framework, isolated large channels, and isolated small channels are shown in **Figure S3d–f**, respectively. The full LUDKOM framework shows a stepwise adsorption process, where water uptake first occurs in the large channels and is followed by a second adsorption step associated with the small ones. The MPD of the isolated large channel shows a gradual transition toward the high-loading state, suggesting a continuous transition. In contrast, the isolated small-channel system exhibits a clear bimodal distribution, indicating that adsorption in the small channels proceeds through a first-order phase transition at a higher pressure.

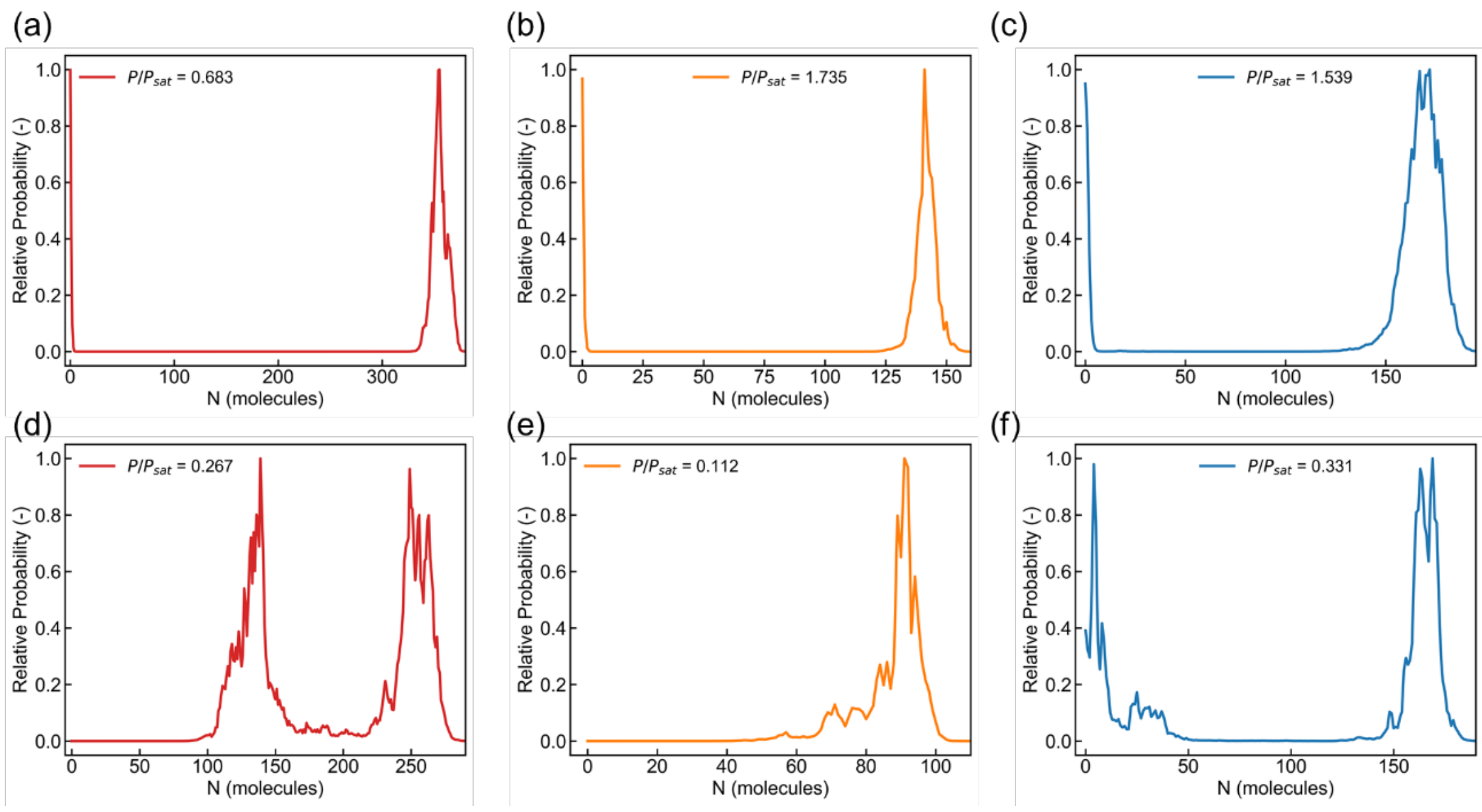

Figure S3. MPD of water adsorption in MOF-806 and LUDKOM systems evaluated near their corresponding adsorption transition pressures. (a–c) MPD for MOF-806, including (a) the full framework, (b) the isolated large pore, and (c) the isolated small pore. The bimodal distributions indicate first-order adsorption transitions in these MOF-806 systems. (d–f) MPD for LUDKOM, including (d) the full framework, (e) the isolated large channels, and (f) the isolated small channels. The large-channel system shows a unimodal macrostate probability distribution, whereas the small-channel system exhibits a bimodal distribution corresponding to a first-order transition at a higher pressure.

6. Comparison of water adsorption and hydrogen-bond formation in the large pore and one small pore

To shed light on the effect of pore size on water adsorption, we further compare the adsorption behavior when only one large pore or only one small pore is accessible. In the former case, only the central large pore is accessible to water, whereas in the latter case, only one of the eight surrounding small pores is accessible. Thus, water adsorption in both cases occurs within a single isolated pore without connections to the surrounding pore network. As shown in **Figure S4a**, water condenses in the large pore at a lower relative pressure than in the small pore. This earlier condensation may be attributed to the greater hydrogen-bond coordination supported by the larger and more bulk-like pore environment. As shown in **Figure S4b**, the average number of hydrogen bonds per water molecule is 3.00 when only one large pore is accessible, compared with 2.85 when only one small pore is accessible. The stronger geometric confinement within the small pore restricts the molecular orientations and configurations available for hydrogen-bond formation, whereas the large pore provides greater configurational freedom and supports a more developed local hydrogen-bond network.

Notably, as also shown in **Figure S4**, substantial water adsorption occurs at an even lower relative pressure in the complete MOF-806 framework than when only one large pore or only one small pore is accessible. Importantly, the complete framework exhibits a higher average hydrogen-bond number of 3.28. This value cannot be explained by a simple weighted average of the contributions from independent large and small pores. Instead, corroborating with the discussion made in the main text, pore connectivity allows water molecules near the cage openings to form hydrogen bonds with molecules in neighboring pore domains, resulting in a more complete and spatially extended hydrogen-bond network.

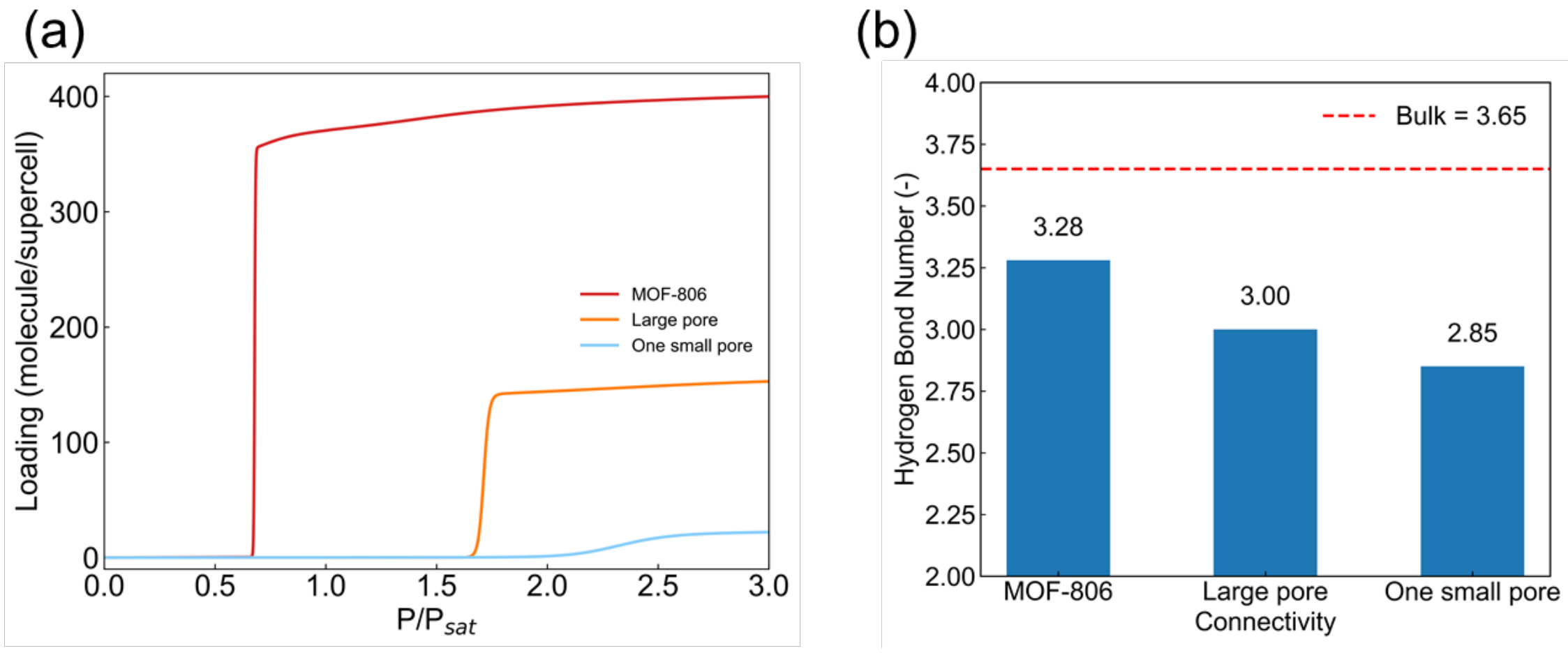

Figure S4. (a) Simulated water adsorption isotherms displaying the loading as a function of relative pressure for the complete MOF-806 framework, the system containing only one accessible large pore, and the system containing only one accessible small pore. (b) Average hydrogen-bond number for the complete framework and the individual pore systems. The dashed line indicates the bulk-water value.

7. Free energy comparison at given relative pressures

Figure S5 compares the free energy profiles of the different connectivity variants at the same relative pressures. At a given pressure, systems with higher pore connectivity exhibit a lower grand potential in the high-loading region relative to the low-loading state, demonstrating that the more connected pore network preferentially stabilizes the condensed water phase. Since phase coexistence occurs when the low- and high-loading minima have equal depths, this stabilization allows the more highly connected systems to reach the coexistence condition at a lower chemical potential and consequently at a lower relative pressure.

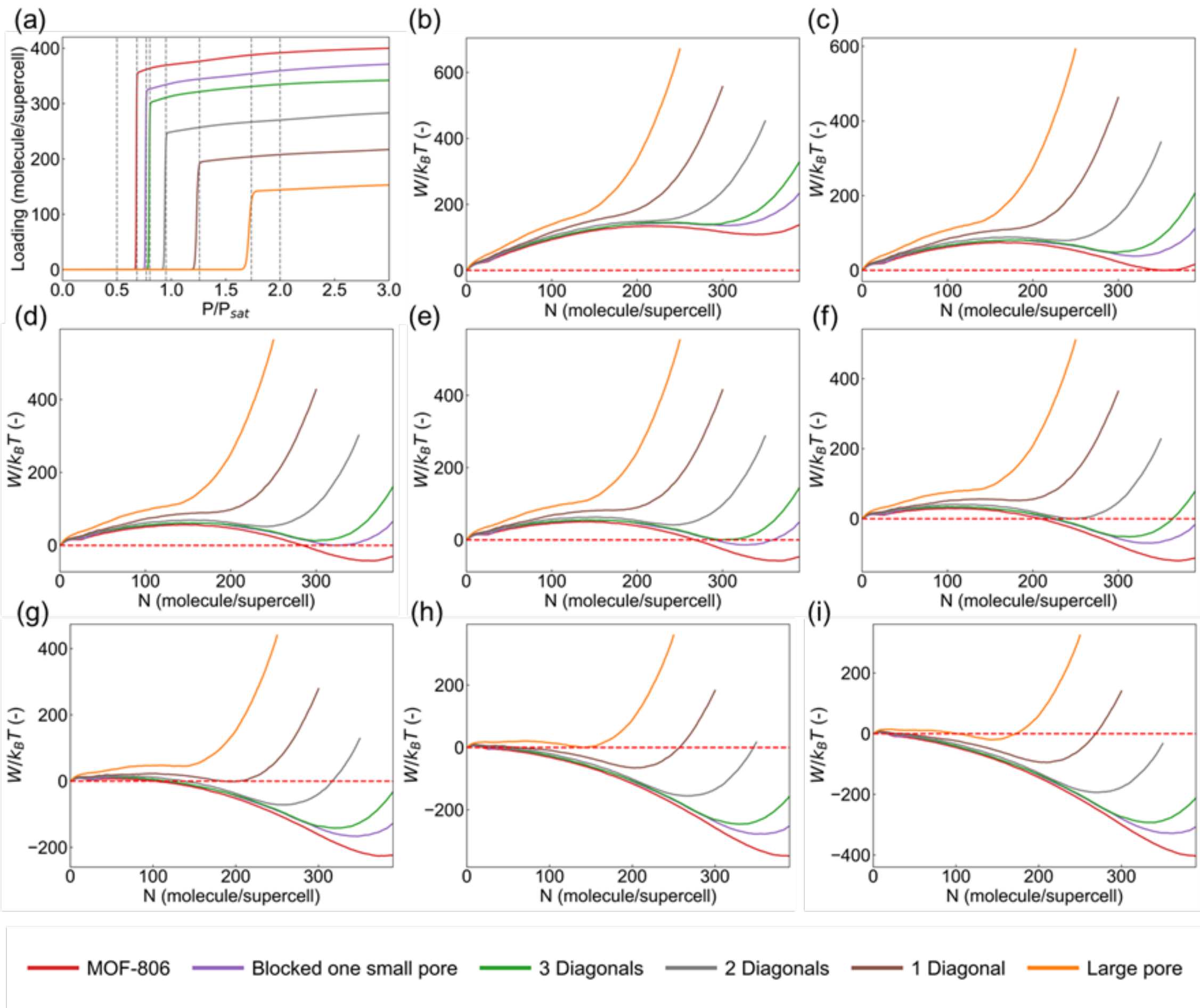

Figure S5. Grand potential free-energy profiles of MOF-806 systems with different pore-connectivity degrees at selected relative pressures. Panel (a) shows the water adsorption isotherms, with vertical dashed lines indicating representative pressures used for the free-

energy comparison. Panels (b)–(i) correspond to the vertical dashed lines in panel (a), arranged from low to high relative pressure: 0.50, 0.68, 0.77, 0.80, 0.95, 1.26, 1.73, and 2.00. The selected pressures include one low-loading condition, the adsorption step pressures of the connectivity-modified systems from high to low connectivity, and one high-loading condition. Each panel shows the corresponding free-energy profile, W/k_BT, as a function of water loading, N.

8. Spatial hydrogen-bonding analyses

To examine the effect of pore connectivity, hydrogen-bond statistics are analyzed as a function of radial distance from the center of the common large pore. As shown in **Figure S6a**, water molecules within the inner region of the large pore exhibit relatively similar average hydrogen-bond numbers among the different connectivity variants. The profiles begin to diverge markedly at radial distances greater than approximately 5 Å, corresponding to the peripheral region of the large pore and the openings toward neighboring small pores. The donor-state distributions exhibit a corresponding spatial dependence, as shown in **Figure S6b–d**. In the central region, DD water remains the dominant species in all structures, and its fraction is relatively insensitive to pore connectivity. Near the pore boundary, however, the DD fraction decreases much more strongly as connectivity is reduced, accompanied by corresponding increases in the SD and ND fractions. The partially blocked structures exhibit intermediate distributions, demonstrating a systematic dependence on the number of accessible neighboring pores.

These results show that pore blocking does not uniformly disrupt the hydrogen-bond network throughout the large pore. Instead, its effect is spatially localized near the cage boundary, where the hydrogen-bond network otherwise extends through the pore openings into neighboring cages. In the connected structures, water molecules near these openings maintain more complete donor coordination through interactions with water in adjacent pore domains. Progressive blocking truncates this network, leading to a pronounced loss of DD water and increased populations of incompletely coordinated SD and ND species. Together with the free-energy results showing greater stability of the high-loading state in the more connected structures, these observations support the role of boundary-spanning hydrogen bonding in stabilizing the condensed adsorbed phase.

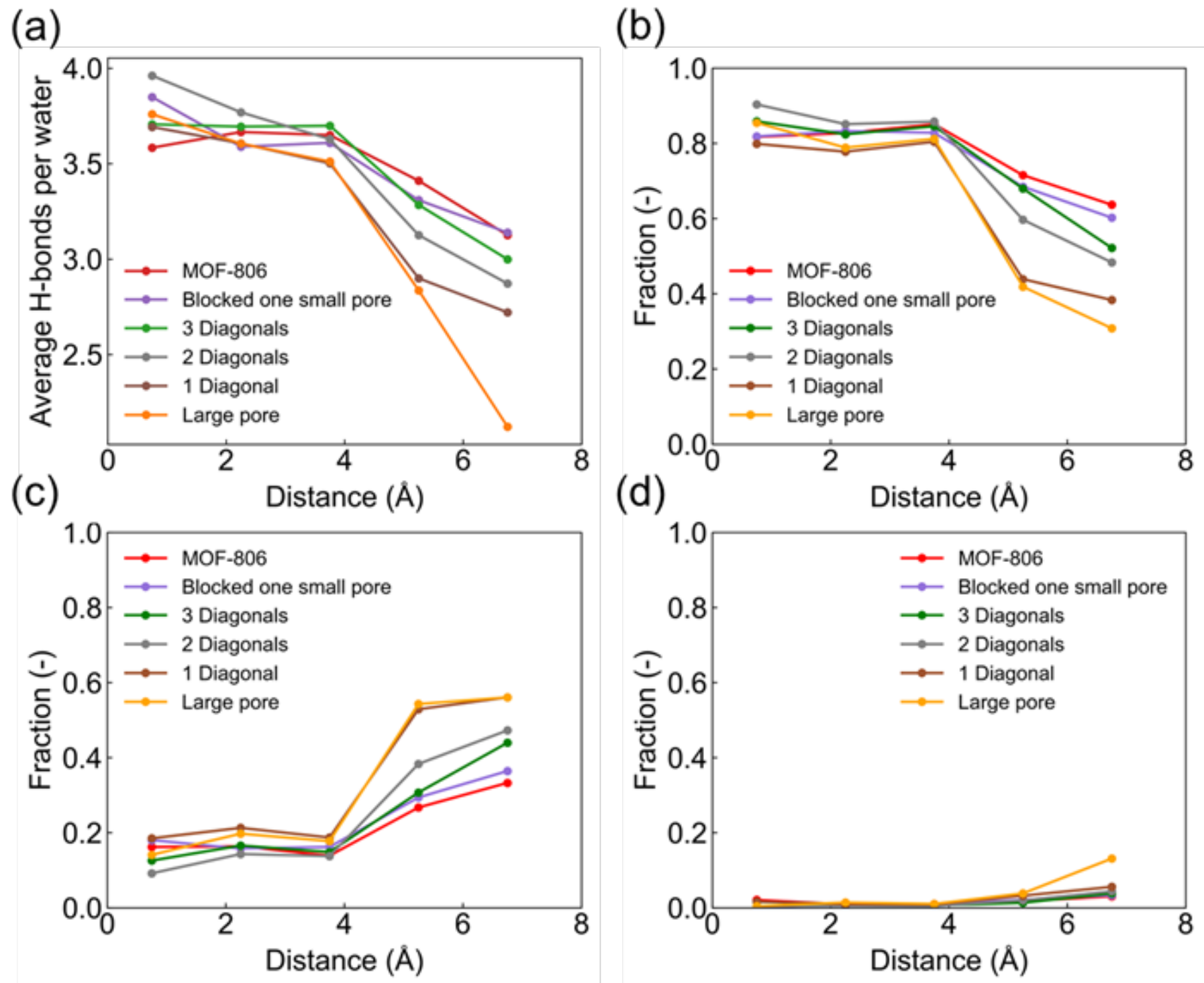

Figure S6. Radial distributions of hydrogen-bonding behavior and water donor configurations in the large pore of MOF-806 under different pore-connectivity conditions. Panel (a) shows the average number of hydrogen bonds per water molecule as a function of the distance from the center of the large pore. Panels (b)–(d) show the corresponding fractions of DD, SD, and ND water molecules, respectively. Different colored lines represent structures with different pore connectivities, ranging from the fully connected MOF-806 framework to the isolated large-pore system.

9. Low-connectivity structure: WOJJOV

WOJJOV is examined as another one-dimensional framework containing spatially independent channels[8]. As shown in **Figure S7a**, the structure consists of two types of channels with pore diameters of approximately 7.8 and 5.8 Å. Water molecules adsorbed in this framework are confined within individual channels, allowing the adsorption behavior of the two channel environments to be analyzed separately. As shown in **Figure S7b**, both the isolated large- and small-channel systems exhibit sharp adsorption steps, indicating that water adsorption in each channel proceeds through a first-order phase transition. For the full WOJJOV framework, the adsorption behavior reflects the combined contribution from these two channel types. Similar to the behavior observed in LUDKOM, the adsorption step of the small channels in the full WOJJOV framework shifts slightly toward lower pressure compared with that in the isolated small-channel system. This again suggests that, although the channels are structurally independent, the adsorption behavior in one channel can still be influenced by the presence of adsorbed water in other channels. To examine whether this shift originates from enhanced hydrogen-bond connectivity between different channels, the average number of hydrogen bonds is calculated for water adsorbed in the full WOJJOV framework and in the corresponding isolated-channel systems. As shown in **Figure S7c**, the average hydrogen-bond number is 3.17 for water adsorbed in the isolated large channel and 2.22 for water adsorbed in the isolated small channel, while the full framework gives an intermediate value of 2.95. This intermediate value mainly reflects the combined contributions from water molecules confined in the two types of channels, rather than the formation of additional hydrogen bonds across different channels. Therefore, consistent with LUDKOM, the shift in adsorption behavior in WOJJOV cannot be primarily attributed to cross-pore hydrogen-bond connectivity. Instead, other intermolecular contributions between adsorbed water molecules in different channels

should be considered.

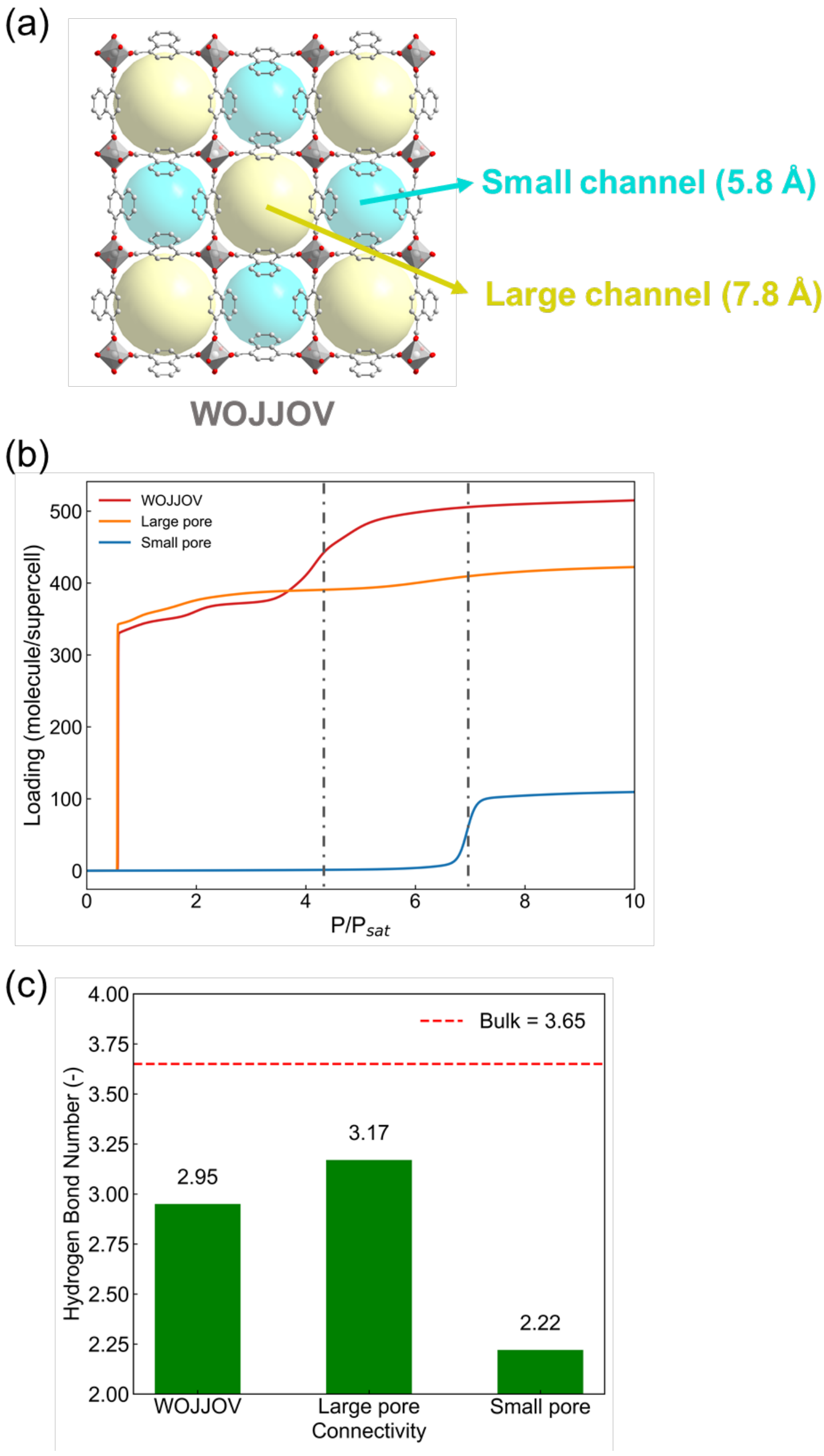

Figure S7. (a) Structure of WOJJOV with one-dimensional channels. The yellow spheres represent the larger channels, and the blue spheres indicate the smaller channels. (b) Simulated water adsorption isotherms displaying the loading as a function of relative pressure for the one-dimensional channel structure WOJJOV. (c) Average hydrogen-bond number for the whole system and individual channel regions.

References

(1) Witman, M.; Mahynski, N. A.; Smit, B. Flat-histogram Monte Carlo as an efficient tool to evaluate adsorption processes involving rigid and deformable molecules. *J. Chem. Theory Comput.* **2018**, *14* (12), 6149–6158. DOI: 10.1021/acs.jctc.8b00534.

(2) Witman, M.; Wright, B.; Smit, B. Simulating enhanced methane deliverable capacity of guest responsive pores in intrinsically flexible MOFs. *J. Phys. Chem. Lett.* **2019**, *10* (19), 5929–5934. DOI: 10.1021/acs.jpclett.9b02449.

(3) Datar, A.; Witman, M.; Lin, L.-C. Improving computational assessment of porous materials for water adsorption applications via flat histogram methods. *J. Phys. Chem. C* **2021**, *125* (7), 4253–4266. DOI: 10.1021/acs.jpcc.0c11082.

(4) Datar, A.; Witman, M.; Lin, L.-C. Monte Carlo simulations for water adsorption in porous materials: best practices and new insights. *AIChE J.* **2021**, *67* (12), e17447. DOI: 10.1002/aic.17447.

(5) Mayo, S. L.; Olafson, B. D.; Goddard, W. A. DREIDING: a generic force field for molecular simulations. *J. Phys. Chem.* **1990**, *94* (26), 8897–8909. DOI: 10.1021/j100389a010.

(6) Rappe, A. K.; Casewit, C. J.; Colwell, K. S.; Goddard, W. A., III; Skiff, W. M. UFF, a full periodic table force field for molecular mechanics and molecular dynamics simulations. *J. Am. Chem. Soc.* **1992**, *114* (25), 10024–10035. DOI:

10.1021/ja00051a040.

(7) Horn, H. W.; Swope, W. C.; Pitera, J. W.; Madura, J. D.; Dick, T. J.; Hura, G. L.; Head-Gordon, T. Development of an improved four-site water model for biomolecular simulations: TIP4P-Ew. *J. Chem. Phys.* **2004**, *120* (20), 9665–9678. DOI: 10.1063/1.1683075.

(8) Comotti, A.; Bracco, S.; Sozzani, P.; Horike, S.; Matsuda, R.; Chen, J.; Takata, M.; Kubota, Y.; Kitagawa, S. Nanochannels of two distinct cross-sections in a porous Al-based coordination polymer. *J. Am. Chem. Soc.* **2008**, *130* (41), 13664–13672. DOI: 10.1021/ja802589u.
